# Modelling of C/Cl isotopic behaviour during chloroethene biotic reductive dechlorination: Capabilities and limitations of simplified and comprehensive models

**DOI:** 10.1371/journal.pone.0202416

**Published:** 2018-08-22

**Authors:** Alice Badin, Fabian Braun, Landon J. S. Halloran, Julien Maillard, Daniel Hunkeler

**Affiliations:** 1 University of Neuchâtel, Centre for Hydrogeology & Geothermics (CHYN), Neuchâtel, Switzerland; 2 Swiss Center for Electronics and Microtechnology (CSEM), Systems Division, Neuchâtel, Switzerland; 3 Ecole Polytechnique Fédérale de Lausanne (EPFL), Signal Processing Laboratory (LTS5), Lausanne, Switzerland; 4 Ecole Polytechnique Fédérale de Lausanne (EPFL), Laboratory for Environmental Biotechnology, Lausanne, Switzerland; Universita degli Studi di Milano-Bicocca, ITALY

## Abstract

Predicting the fate of chloroethenes in groundwater is essential when evaluating remediation strategies. Such predictions are expected to be more accurate when incorporating isotopic parameters. Although secondary chlorine isotope effects have been observed during reductive dechlorination of chloroethenes, development of modelling frameworks and simulation has thus far been limited. We have developed a novel mathematical framework to simulate the C/Cl isotopic fractionation during reductive dechlorination of chloroethenes. This framework differs from the existing state of the art by incorporating secondary isotopic effects and considering both C and Cl isotopes simultaneously. A comprehensive general model (GM), which is expected to be the closest representation of reality thus far investigated, was implemented. A less computationally intensive simplified model (SM), with the potential for use in modelling of complex reactive transport scenarios, was subsequently validated based on its comparison to GM. The approach of GM considers all isotopocules (i.e. molecules differing in number and position of heavy and light isotopes) of each chloroethene as individual species, of which each is degraded at a different rate. Both models GM and SM simulated plausible C/Cl isotopic compositions of tetrachloroethene (PCE), trichloroethene (TCE) and *cis*-1,2-dichloroethene (*c*DCE) during sequential dechlorination when using experimentally relevant kinetic and isotopic parameters. The only major difference occurred in the case where different secondary isotopic effects occur at the different non-reacting positions when PCE is dechlorinated down to *c*DCE. This observed discrepancy stems from the unequal Cl isotope distribution in TCE that arises due to the occurrence of differential secondary Cl isotopic effects during transformation of PCE to TCE. Additionally, these models are shown to accurately reproduce experimental data obtained during reductive dechlorination by bacterial enrichments harbouring *Sulfurospirillum* spp. where secondary isotope effects are known to have occurred. These findings underscore a promising future for the development of reactive transport models that incorporate isotopic parameters.

## Introduction

Owing to recent analytical method developments [1–5] and to numerous studies demonstrating the potential of applications of compound specific isotopic analysis (CSIA) in estimating the origin and fate of chlorinated solvents in the subsurface [6–8], environmental samples are increasingly being submitted for isotopic analysis. Previous studies showed that such analysis can facilitate the determination of the extent of biodegradation in groundwater based on isotopic enrichment factors determined via the Rayleigh equation. This is possible due to the fact that molecules containing different proportions of isotopes of one element (i.e., isotopologues) follow different reaction kinetics. This is caused by the different energy required to break the chemical bonds binding elements with heavy or light isotopes which eventually induces the observed variation in isotopic composition during biodegradation.

An accurate simulation of the isotopic ratio evolution during biodegradation may allow a better integration of isotopic data when estimating the fate of a plume undergoing natural attenuation. Several studies have been carried out to address this challenge, some of which additionally successfully applied models to evaluate the fate of chloroethenes plumes [9–13]. However, for simplification purposes, most models considered one element only [9, 11, 14, 15], and some models simulated heavy and light isotopes of one element of each compound as separate species [14, 15] while others considered isotopologues with regards to one [11, 16] or two elements [13]. Such a variety of simplification types reflects the need for a trade-off between including all isotopologues or isotopocules (i.e., isotopomers of all isotopologues of one compound; isotopomers being isomers of isotopologues) and avoiding an excessive complexity leading to computationally demanding models. More specifically, Jin et al. [13] demonstrated that considering simultaneously combined C-Cl isotopologues could change simulated isotopic behaviours at late reaction times and for large differences between the C and Cl enrichment factors compared to the simplified method developed by Hunkeler et al. [11] where Cl isotopologues/isotopes only were considered. However, secondary isotope effects, which affect elements located in non-reacting bonds, were neglected in this work whichalso ignored PCE. Recent studies have shown that secondary Cl isotopic effects are measurable during chloroethene reductive dechlorination and should therefore not be discounted [16–18]. However, few models so far have considered secondary isotopic effects since they were formerly generally assumed to be negligible [13, 19, 20]. To address this need, Höhener [18] and van Breukelen et al. [16] recently incorporarted secondary isotopic effects in isotope fractionation models though both studies considered isotopes of all elements independantly. Van Breukelen et al. [16] applied a correction term to the reaction rate of each isotope/isotopologue so that the independent isotope networks correspond to the overall reaction progress. On the other hand, Höhener et al. applied a “Cretnik correction” which involved the introduction of an offset. Both successfully tested their models against TCE reductive dechlorination data [17, 21].

To the best of our knowledge, no model without simplification that considers whole molecules as they are in reality (i.e. not considering isotopes of elements separately) has thus far been developed. Such a model might (i) enable determination of the limits of simplified models by comparing its outcome with that of a simplified model and (ii) allow for the development of models for other compounds or even for the incorporation of clumped isotope effects. Additionally, former work has focused on the reductive dechlorination of chloroethenes starting with TCE.

Concurrently considering (i) Monod kinetics, (ii) secondary isotopic effects, and (iii) several elements simultaneously and the corresponding isotopocules to which they belong is a challenging and important task in the simulation of the evolution of chlorinated ethenes isotopic composition during reductive dechlorination. Addressing this knowledge gap may help with the integration of isotopic data from field sample measurements in reactive transport models for improved plume fate prediction. To this end, the present contribution proposes a generic isotope modelling framework which is more comprehensive than the current state of the art. With a focus on reductive dechlorination of chloroethenes, this study is focused on: (i) developing and implementing a novel comprehensive and generic general model (GM) which allows simulation of the simultaneous evolution of isotopic composition in chloroethenes during sequential reductive dechlorination considering Monod kinetics and secondary isotopic effects, (ii) setting up a computationally less demanding simplified model (SM) matching the general model which meets the same requirements with regards to Monod kinetics and secondary isotopic effects, (iii) verifying whether this model can accurately reproduce C and Cl isotope data obtained during reductive dechlorination of chloroethenes by two different bacterial consortia, and (iv) identify where the two models diverge in predicting isotopic enrichment/depletion. Finally, this work also seeks to provide the community with a relevant software repository to encourage the integration of primary and seconday isotope effects into future modeling scenarios.

Details of the mathematical framework of the models are first presented. This is followed by a description of the implementation, evaluation, and comparison of the GM and SM; an assessment of the feasibility of fitting these models to experimental data; and finally, a discussion of the significance for the assessment of sites contaminated by chloroethenes.

## Mathematical model development

The general (GM) and simplified (SM) models developed here to simulate isotope trends take into account primary and secondary isotope effects as well as Monod kinetics. Isotopocules designate all isotopomers of all isotopologues, i.e. the entire set of molecules of the same compound differing both in the number and position of light and heavy isotopes.

### General model (GM): Simultaneous consideration of C and Cl isotopes

#### General expression

Traditionally, fractionation between isotopes rather than between isotopocules has been considered [22]. Such fractionation is described by the kinetic isotope fractionation factor α_k_ relating the isotope ratio of the instantaneous product to the isotope ratio of the substrate. The difference in rate transfer from reactant to product pool associated with heavy and light isotopes, ^*H*^*k*_bulk_ and ^*L*^*k*_bulk_, respectively, can also be described by α_k_ which equals the ratio ^*H*^*k*_bulk_/^*L*^*k*_bulk_ [22]. In order to incorporate isotope fractionation during sequential dechlorination in a reactive transport model, Van Breukelen et al. [14] previously established the following expression relating the ^13^C isotope reaction rate ^13^*r*_*S*_ of a consumed substrate (S) to overall reaction rate *r*_*S*_ of S:

$${}^{13}r_{S}=r_{S}\frac{{[}^{13}S]}{\left[S\right]}\alpha_{k}$$ (1)

This equation is valid under the assumption that the degradation rate of the predominant ^12^C isotope corresponds to the overall degradation rate of S corrected for the proportion of ^12^C to total carbon [14]. The terms [^13^*S*] and [*S*] correspond to the ^13^C concentration in S and the total S concentration, respectively. A similar approach is applied in the present work to simulate the evolution of a compound’s isotopic composition considering simultaneously all elements affected by isotopic fractionation and including secondary isotope effects. In this case, instead of two isotopes, each isotopocule is considered as an individual entity undergoing reaction at a specific rate related to its isotopic composition and therefore to the “isotopocule fractionation” it undergoes. We thus expand the expression suggested by Van Breukelen et al. [14] for isotopocules of both produced and degraded compounds. More particularly, we ensurethat a given isotopocule of a produced compound is yielded by specific isotopocules of its precursor compound and thus appears in the expanded expression.

**Fig 1:**
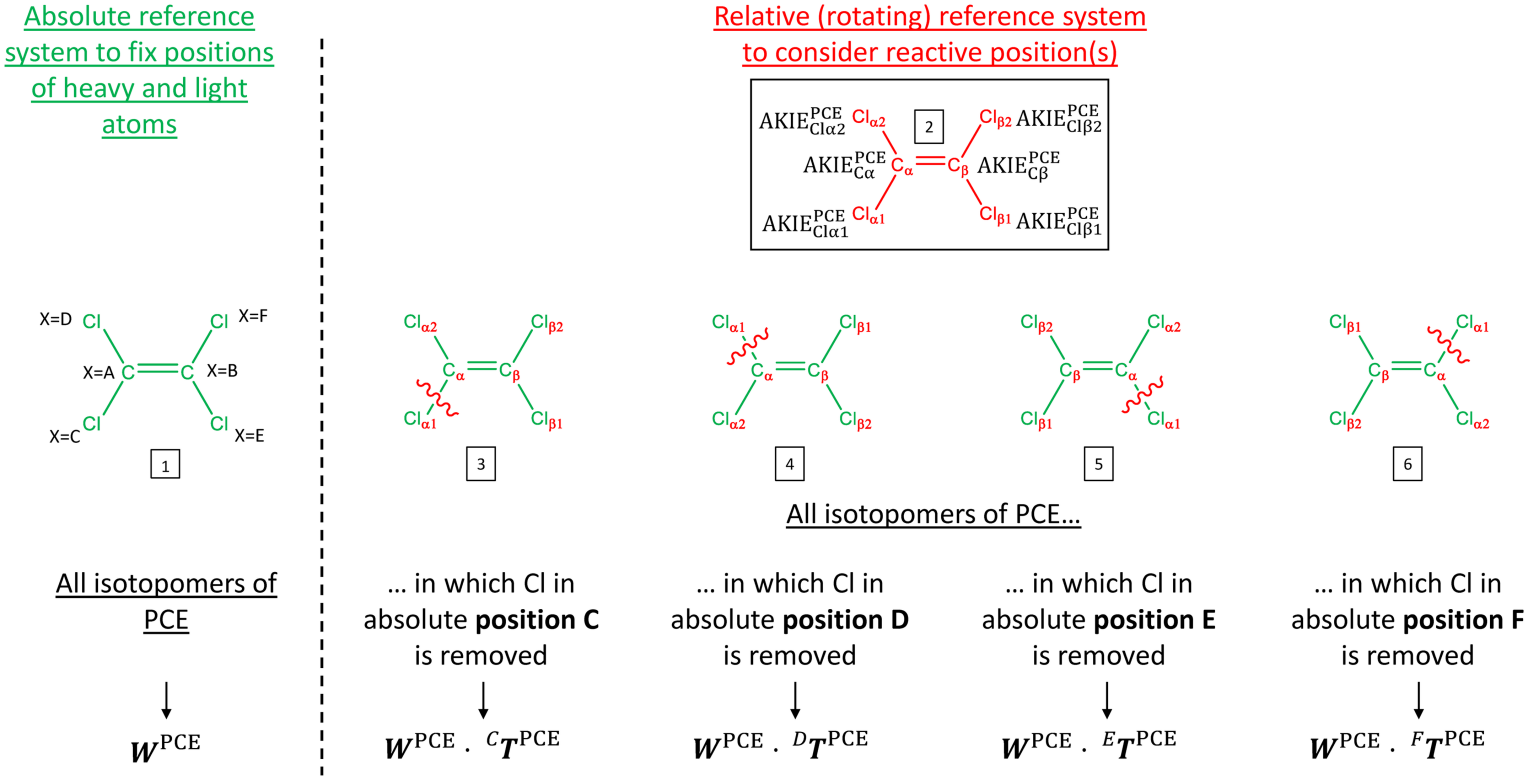
Absolute and relative reference systems used to determine weight coefficients associated with AKIE_p_ for further calculation of isotopocule fractionation factors. Molecule 1 illustrates the absolute reference system where each of the 6 atoms adopt absolute positions and based on which all 64 isotopocules of PCE are determined. This absolute reference system is described by ***W***^PCE^. Molecule 2 illustrates the relative reference system based on which each reactive bond can be distinctly considered. Molecules 3 to 6 illustrate the result of transformation of the absolute system with a rotating reference system required to determine position-specific AKIEs related to the removed atom at absolute position X = C, E, F, and D, respectively. Cl_α1_ will typically undergo a primary Cl isotopic effect while Cl_α2_, Cl_β1_ and Cl_β2_ will undergo secondary Cl isotopic effects. Similarly, C_α_ will undergo a primary C isotopic effect and C_β_ a secondary C isotopic effect. The green color indicates the absolute reference system; the red color indicates the relative reference system.

**Fig 2:**
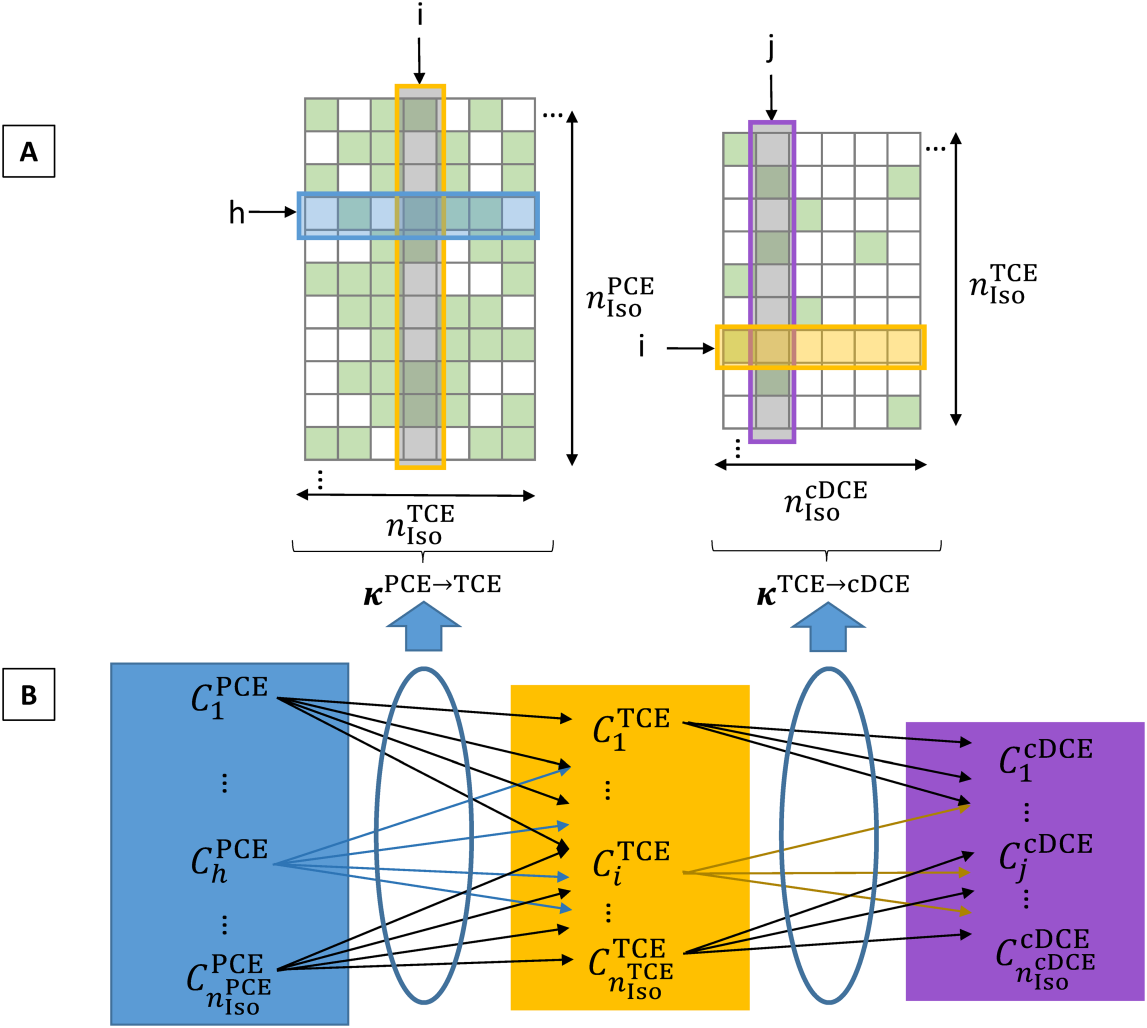
Schematic explanation of matrices *κ*^PCE→TCE^ ($\in R^{64\times 32}$) and *κ*^TCE→*c*DCE^ ($\in R^{32\times 16}$) containing kinetic isotopic coefficients $\kappa_{h,i}^{PCE\to TCE}$ and $\kappa_{i,j}^{TCE\to cDCE}$, associated with reductive dechlorination of PCE to TCE and TCE to *c*DCE, respectively. Panel A: For the sake of simplicity, smaller matrices than the actual ones used are represented here. The green and white squares represent isotopocule fractionation factors different from zero and equal to zero, respectively. Columns i and j correspond to the column of factors associated with the production of the i^th^ isotopocule of TCE from PCE and the j^th^ isotopocule of *c*DCE from TCE, respectively. The blue shade in panel A corresponds to the factors associated with degradation of the h^th^ isotopocule of PCE to TCE which are further illustrated by the blue arrows in panel B. Similarly, the orange shade in panel A corresponds to the factors associated with degradation of the i^th^ isotopocule of TCE to *c*DCE which are illustrated by the orange arrows in panel B.

The reaction rate of each isotopocule *i* of intermediate (i.e. both produced and degraded) compound γ resulting from the degradation of isotopocules *h* of compound γ-1 and being further degraded to isotopocules *j* of compound γ+1 can then be described by the following general equation (note that the right-most term describes the rate of degradation to γ+1 and the preceeding term describes the rate of production from γ-1):

$$\frac{\partial C_{i}^{\gamma }}{\partial t}=r_{i}^{\gamma }=\left(\sum_{h=1}^{n_{\text{Iso}\;}^{\gamma -1}}\kappa_{h,i}^{\gamma -1\to \gamma }\cdot v^{\gamma -1}\frac{C_{h}^{\gamma -1}}{C_{\text{tot}}^{\gamma -1}}\right)-\left(\sum_{j=1}^{n_{\text{Iso}\;}^{\gamma +1}}\kappa_{i,j}^{\gamma \to \gamma +1}\right)\cdot v^{\gamma }\frac{C_{i}^{\gamma }}{C_{\text{tot}}^{\gamma }}$$ (2)

$$\forall i\in [1,\ldots ,n_{Iso}^{\gamma }]$$

where *C*_*i*_ and *C*_*tot*_ are, respectively, the isotopocule *i* and total compound concentrations (i.e. $C_{tot}^{\gamma }=\sum_{i=1}^{n_{Iso}^{\gamma }}C_{i}^{\gamma }$ and $C_{tot}^{\gamma -1}=\sum_{h=1}^{n_{Iso}^{\gamma -1}}C_{h}^{\gamma -1}$ where $n_{Iso}^{\gamma }$ and $n_{Iso}^{\gamma -1}$ are the number of isotopocules of compound γ and γ-1), *r* is the isotopocule reaction rate, *v* is the total compound reaction rate. The matrix ***κ*** describes the difference in isotopocule reaction rates due to the presence of light or heavy atoms in reacting (primary isotope effect) and/or non-reacting positions (secondary isotope effect). It is hence analogous to *α*_*k*_, except that it applies to isotopocules instead of isotopes. It theoretically allows any transition from one isotopocule of a degraded compound (i.e., breaking any reactive bond) to any isotopocule of the compound it produces and, as such, is fully general. For chloroethenes in particular, there are only a few possible transitions from isotopocules of the degraded compound to isotopocules of the produced compound (e.g., maximum 4 transitions for 1 PCE isotopocule degraded to TCE), thus the matrix is sparse, i.e., mostly filled with zero elements which exclude non-existing transitions. The non-zero elements of the matrix represent the isotopocule fractionation factors. Here $\kappa_{h,i}^{\gamma -1\to \gamma }$ thus represents an element of the isotopocule fractionation matrix $\kappa_{}^{\gamma -1\to \gamma }(\in R^{n_{Iso}^{\gamma -1}\times n_{Iso}^{\gamma }}$) containing kinetic isotopocule fractionation factors associated with isotopocules h of compound γ-1 leading to isotopocule i of compound γ. Analogously, the matrix $\kappa_{\;}^{\gamma \to \gamma +1}(\in R^{n_{Iso}^{\gamma }\times n_{Iso}^{\gamma +1}}$) describes the transitions from compound γ leading to compound γ+1. ***κ*** is more specifically defined as:

κh,iγ−1→γ={1nRBγ−1∏p=1nAtomsγ−1(1AKIEpγ−1)(Wγ−1⋅TXγ−1)h,p,ifremovingatomatabsolutepositionXofisotopoculeh(ofcompoundγ-1)leadstoisotopoculei(ofcompoundγ)0,else (3)

where γ and γ-1 designate the produced and degraded compounds, respectively, and h and i the isotopocules of γ and γ-1, respectively. *n*_*RB*_ is the number of reactive bonds, AKIE_*p*_ the position-specific apparent kinetic isotopic effect associated with the atom at relative position p when removing the atom at absolute position X. The weight coefficient matrix ***W***^*γ*-1^ ($\in R^{n^{\gamma -1}\times n_{Atoms}^{\gamma -1}}$) accounts for all possible combinations of heavy (weight = 1) and light (weight = 0) isotopes in the molecule and thus describes all possible isotopocules. For each of the possible bond breakage positions (removal of atom X) a transformation matrix ^***X***^***T***^*γ*-1^ ($\in R^{n_{Atoms}^{\gamma }\times n_{Atoms}^{\gamma }}$) exists, which transforms W from an absolute reference system (topologically fixed) to a relative reference system (relative to the bond breakage position) as illustrated for PCE in Fig 1. This transformation step is necessary as the vector AKIE is arranged in a relative reference system. To clarify these concepts, two examples of the ***κ*** matrix associated with PCE reductive dechlorination to *c*DCE (***κ***^PCE→TCE^ and ***κ***^TCE→*c*DCE^) are illustrated in Fig 2.

The product in each non-zero element of ***κ*** takes into account the position-specific apparent kinetic isotopic effects (AKIEs) associated with each atom of the isotopocule of the degraded compound. The latter accounts for primary and secondary isotopic effects in isotopocule fractionation factors and is defined as follows:

$${\text{AKIE}}^{\gamma }=\left[\frac{1}{1+\frac{\varepsilon_{1}}{1000}},\;\ldots ,\frac{1}{1+\frac{\varepsilon_{n_{\text{Atoms}}^{c}}}{1000}}\right]\in {\mathbb{R}}^{n_{\text{Atoms}}^{\gamma }}$$ (4)

where *ε*_*p*_ (with $p\in [1,\ldots ,n_{Atoms}^{\gamma }]$) corresponds to the degradation-related position-specific isotopic effect (either primary or secondary) of the atom at relative position p.

Additionally, clumped isotope effects, where the isotope effect associated with the presence of a heavy atom at a certain position depends on the presence of heavy atoms at other positions, could be included in order to have a more comprehensive model. They were however not included when determining ***κ*** due to the lack of experimental evidence for the occurrence of such effects in the case of chloroethenes reductive dechlorination and the challenges of their potential measurement. Yet, as each isotopocule is considered separately, the approach could easily be adapted to include clumped isotope effects should future advances enable their measurement. The only modification necessary would consist of adapting the non-zero elements of ***κ***.

#### Application of GM to PCE reductive dechlorination

First, the general concept of GM applied to the reductive dechlorination of PCE to *c*DCE is presented followed by a more detailed explanation of how the matrix ***κ*** is generated for the PCE to TCE transformation step. Fig 2B illustrates the sequential reductive dechlorination of PCE isotopocules to TCE and consecutively to *c*DCE isotopocules. Fig 2A illustrates the corresponding isotopocule fractionation matrices $\kappa_{\;}^{PCE\to TCE}\in R^{64\times 32}$ and $\kappa_{\;}^{TCE\to cDCE}\in R^{32\times 16}$. The number of lines in each matrix corresponds to the number of isotopocules of the degraded compound. Similarly, the number of columns in each matrix corresponds to the number of isotopocules of produced compound. In each line corresponding to the degradation of one isotopocule, each non-zero element corresponds to the isotopocule fractionation factor associated with each breakable bond position. In the case of PCE to *c*DCE reductive dechlorination, a total number of $n_{Iso}^{PCE}=2^{6}=64$ (6 atoms, each having two possible states, i.e. either a heavy or a light isotope) isotopocules of PCE each yield 4 possible TCE isotopocules (= 4 breakable bonds) among a total number of $n_{Iso}^{TCE}=2^{5}=32$ isotopocules of TCE. In ***κ***^PCE→TCE^, each line will thus contain 4 non-zero elements corresponding to the different possible bond breakage positions. Each TCE isotopocules will in turn yield 1 *c*DCE isotopocule (= 1 breakable bond) among a total number of $n_{Iso}^{cDCE}=2^{4}=16$ isotopocules of *c*DCE. Similarly, in ***κ***^TCE→*c*DCE^, each line will thus contain 1 non-zero element corresponding to the different possible bond breakage positions. It should be noted that different isotopocules of degraded compound may yield the same isotopocule of produced compound. Furthermore, for symmetric molecules such as PCE and *c*DCE, the total number of isotopocules may be reduced due to symmetries.

PCE reductive dechlorination to TCE (ignoring subsequent degradation to *c*DCE), denoted as PT, is considered here to illustrate the application of Eq (2). As PCE is being degraded, the concentration of its isotopocules h will thus follow:

$$\frac{\partial C_{h}^{\text{PCE}}}{\partial t}=-\left(\sum_{i=1}^{n_{\text{Iso}}^{\text{TCE}}}\kappa_{h,i}^{\text{PCE}\to \text{TCE}}\right)\cdot v^{\text{PCE}}\frac{C_{h}^{\text{PCE}}}{C_{\text{tot}}^{\text{PCE}}},\;\forall h\in \left[1,\ldots ,n_{\text{Iso}}^{\text{PCE}}\right]$$ (5)

Conversely, the concentration of isotopocules i of produced TCE will follow:

$$\frac{\partial C_{i}^{\text{TCE}}}{\partial t}=\sum_{h=1}^{n_{\text{Iso}}^{\text{PCE}}}\left(\kappa_{h,i}^{\text{PCE}\to \text{TCE}}\cdot v^{\text{PCE}}\frac{C_{h}^{\text{PCE}}}{C_{\text{tot}}^{\text{PCE}}}\right),\;\forall i\in \left[1,\ldots ,n_{\text{Iso}}^{\text{TCE}}\right]$$ (6)

These equations are based on the general Eq (2). Equations (S1), (S2) and (S3) describing PCE, TCE and *c*DCE isotopocules during sequential reductive dechlorination are included in S1 File (Supporting Information). In order to generate ***κ***^PCE→TCE^, the weight coefficient matrix ***W***^PCE^ ($\in R^{64\times 6}$) is created to describe the position of heavy (weight = 1) and light (weight = 0) isotopes in all degraded PCE isotopocules by means of an absolute reference system where the position of all atoms in the molecule are “topologically” fixed (Fig 1, molecule 1). Each of the 64 lines in ***W***^PCE^ represents an isotopocule whereas each of the 6 columns corresponds to one atom at absolute position X being light or heavy. This matrix is then transformed into the aforementioned relative reference system by using one of the four transformation matrices ^***C***^***T***^PCE^, ^***D***^***T***^PCE^, ^***E***^***T***^PCE^, or ^***F***^***T***^PCE^ enabling the correct treatment of a broken bond at absolute position X = C, D, E, or F, respectively (Fig 1, molecules 3 to 6).

The AKIEs themselves are given in the relative system (Fig 1, molecule 2) and correspond to the kinetic isotopic effects associated with each atom at relative position p ∈ [1,…,6]. Typically, AKIE = [AKIE_Cα_, AKIE_Cβ_, AKIE_C1α1_, AKIE_C1α2_, AKIE_C1β1_, AKIE_C1β2_], where C_α_ undergoes a primary C isotopic effect and C_β_ a secondary C isotopic effect; Cl_α1_ undergoes a primary Cl isotopic effect while Cl_α2_, Cl_β1_ and Cl_β2_ undergo secondary Cl isotopic effects. Thanks to the transformed weight coefficient matrix ***W***^PCE^·^***X***^***T***^PCE^, these AKIEs will be accounted for (weight = 1) or not (weight = 0) in ***κ***^PCE→TCE^ depending on whether a heavy or a light isotope is present in the absolute position X as described earlier in Eq (3).

### Separate consideration of C and Cl isotopes (SM)

In view of incorporating the change of isotopic ratios during degradation in a reactive transport model, for example, the general model (GM) was simplified to a less computationally expensive simplified model (SM). This simplification was achieved by simulating fewer species simultaneously.

This model as applied to chloroethenes is explained here and is illustrated in Fig A in S1 File. First, C and Cl atoms are considered separately. Second, as the bond cleavage can take place at any position involving a Cl atom (non-regioselective reaction) for symmetric chloroethenes (e.g. PCE), the positions of heavy and light Cl and C isotopes in the molecule are no longer relevant and we thus consider isotopologues relative to C and Cl instead of isotopocules. Since no differentiation is made between the secondary positions in this model, a single secondary isotopic effect (${\text{AKIE}}_{\text{ClSec}}^{\gamma }$) is considered which reflects all position-specific secondary isotopic effects associated with all Cl atoms located in remaining positions (e.g. for PCE: ${\text{AKIE}}_{\text{Cl}\alpha 2}^{\text{PCE}}={\text{AKIE}}_{\text{Cl}\beta 1}^{\text{PCE}}={\text{AKIE}}_{\text{Cl}\beta 2}^{\text{PCE}}={\text{AKIE}}_{\text{ClSec}}^{\text{PCE}}$). This assumption was made according to the explanations of Cretnik et al. [17] who showed that the overall secondary isotopic effect corresponds to the average between all secondary isotopic effects. Finally, a distinction in treatment is made between symmetric and asymmetric molecules.

Based on these considerations, the corresponding fractionation factor associated with Cl thus reflects (i) both the isotopic effects induced when cleaving a bond involving a heavy Cl isotope (primary isotopic effect) and the isotopic effect induced by the presence of heavy Cl isotopes in the remaining positions which do not react (secondary isotopic effect) (first condition of ***κ***^*γ*→*γ*+1^ described in Eq (7)) or (ii) the isotopic effects induced only by the presence of heavy Cl isotopes in the positions which do not react when cleaving a bond involving a light Cl isotope (second condition of ***κ***^*γ*→*γ*+1^ described in Eq (7)).

Isotopologue fractionation factors relative to Cl are hence calculated as follows:

κi,jγ→γ+1={1AKIEClPrimγ⋅(1AKIEClSecγ)n37Cliγ−1⋅(n37CliγnClγ),ifbondbreakageinvolvingheavyisotopeinisotopologueileadstoisotopologuej(γ+1)(1AKIEClSecγ)n37Cliγ⋅(1−n37CliγnClγ),ifbondbreakageinvolvinglightisotopeinisotpologueileadstoisotopologuej(γ+1)0,else (7)

Contrary to Cl, C atoms are not removed during the reaction, isotopologue fractionation factors associated with C were hence determined as follows:

$$\kappa_{i,j}^{\gamma \to \gamma +1}=\{\begin{array}{rr} \frac{1}{{\text{AKIE}}_{\text{CPrim}}^{\gamma }}\cdot {\left(\frac{1}{{\text{AKIE}}_{\text{CSec}}^{\gamma }}\right)}^{n_{13{\text{C}}_{i}}^{\gamma }-1}\cdot \left(\frac{n_{13{\text{C}}_{i}}^{\gamma }}{n_{\text{C}}^{\gamma }}\right) & \\ +{\left(\frac{1}{{\text{AKIE}}_{\text{CSec}}^{\gamma }}\right)}^{n_{13{\text{C}}_{i}}^{\gamma }}\cdot \left(1-\frac{n_{13{\text{C}}_{i}}^{\gamma }}{n_{\text{C}}^{\gamma }}\right), & \begin{array}{c} \text{if}\;\text{isotopologue}\;\text{i}\\ \text{leads}\;\text{to}\;\text{isotopologue}\;\text{j} \end{array}\\ 0, & \text{else} \end{array}$$ (8)

where i and j correspond to isotopologues of compound γ and γ+1. The specific matrices ***κ*** containing isotopologue fractionation factors associated with sequential reductive dechlorination of PCE to *c*DCE are given in Fig B in S1 File. These specific matrices ***κ*** differ slightly from their definitions in Eqs (7) and (8) as they are further modified to meet the requirements for asymmetric molecules as explained in the following.

Contrary to symmetric molecules, the bond cleavage is regioselective for asymmetric chloroethenes (e.g. TCE). For isotopologues containing both heavy and light Cl isotopes, the positions thereof should therefore be taken into account so that the fact that the bond breakage takes place only where the isotope located in the only reactive position may be considered. “Isotopocules” relative to Cl were hence considered in the case of asymmetric chloroethenes instead of isotopologues. The fractionation factors were determined similarly as for symmetric molecules with the exception that instead of considering any Cl position, the presence of heavy or light Cl isotope in the only possible cleaved position (α1) was taken into account separately from heavy or light Cl isotopes located in non-reacting positions (Figs A and B in S1 File). In the case of C which constitutes the backbone during sequential dechlorination, the probability that a heavy or a light atom is involved in the bond breakage is considered equal when both a light and a heavy isotope are present in the molecule for symmetric molecules (e.g. PCE). Conversely for asymmetric molecules (e.g. TCE), since only one C is involved in the bond-breakage in one isotopocule, the occurrence of primary and secondary isotopic effect is not distributed between the two positions as illustrated for the case of TCE ($\kappa^{{TCE}_{C}\to {cDCE}_{C}}$) (Fig B in S1 File).

### The case of Monod kinetics

The bacterial growth on sequential or simultaneous substrates following Monod kinetics can be described as by Kompala et al. [23]:

$$\frac{\partial X}{\partial t}=\sum_{\gamma =1}^{n_{\text{Comps}}-1}\left(\mu_{\text{MAX}}^{\gamma }\frac{X\cdot C_{\text{tot}}^{\gamma }}{K_{m}^{\gamma }+C_{\text{tot}}^{\gamma }}\right)-\mu_{\text{DEC}}\cdot X$$ (9)

where *n*_Comps_ is the total number of compounds used for growth, X [g of protein·L^-1^] is the biomass concentration growing on all compounds, *C*_tot_ [μmol·L^-1^] is the concentration, μ_MAX_ [μmol·g of protein^-1^·s^-1^] is the maximum growth rate, *K*_*m*_ [μmol·L^-1^] is the half saturation constant, and μ_DEC_ [s^-1^] is the biomass decay rate constant associated with growth on chloroethenes. As the isotopic shifts triggered by compound consumption underlie the primary interests of this study, the biomass growth phase is our focus. The biomass decay rate constant (μ_DEC_) associated with growth is thus assumed to be zero here.

If Monod kinetics are assumed, the following expression for isotopocules (respectively isotopologues for SM) rates may be expressed based on Eq (2), considering growth on sequential or simultaneous substrates where substrates correspond to isotopocules:

$$\frac{\partial C_{i}^{\gamma }}{\partial t}=\left(\sum_{h=1}^{n_{\text{Iso}}^{\gamma -1}}\kappa_{h,i}^{\gamma -1\to \gamma }\cdot \frac{\mu_{\text{MAX}}^{\gamma -1}\cdot X\cdot C_{h}^{\gamma -1}}{Y^{\gamma -1}\cdot \left(K_{m}^{\gamma -1}+C_{\text{tot}}^{\gamma -1}\right)}\right)-\left(\sum_{j=1}^{n_{\text{Iso}}^{\gamma +1}}\kappa_{i,j}^{\gamma \to \gamma +1}\right)\cdot \frac{\mu_{\text{MAX}}^{\gamma }\cdot X\cdot C_{i}^{\gamma }}{Y^{\gamma }\cdot \left(K_{m}^{\gamma }+C_{\text{tot}}^{\gamma }\right)}$$ (10)

$$\forall i\in [1,\ldots ,n_{Iso}^{\gamma }]$$

where Y [g of protein·μmol of released chloride^-1^] represents the biomass yield. The first term corresponds to the production of compound γ from γ-1 and the second term to its degradation to γ+1.

Equations developed for PCE reductive dechlorination to *c*DCE are given in S1 File.

### Initial isotopocules/isotopologues concentrations and final isotopic compositions

The initial isotopocules (GM) and isotopologues (SM) concentrations as well as the final C and Cl isotopic compositions were determined as suggested by Jin et al. [13] and Hunkeler et al. [11] and are described in S1 File.

## Model implementation, evaluation and comparison

### Implementation method

Both GM and SM models were applied to simulate dechlorination of PCE to TCE (PT), PCE to *c*DCE with TCE accumulation (PTD), and TCE to *c*DCE (TD). Simulations with GM were performed with Matlab while simulations with SM were performed both with Matlab and COMSOL Multiphysics (refer to S1 File for the differential equations used in COMSOL and to S2 File for the documented modelling files used in this study). Considering TCE dechlorination to *c*DCE (i.e. in TD and PTD simulations), we assume that the Cl positions in TCE are strictly distinguished between reactive and non-reactive positions. The *c*DCE Cl isotopic composition thus reflects the secondary isotopic effect only [17]. For simplification purposes, it is also assumed that no selective interconversion of *cis*/*trans*-DCE intermediates occur.

**Fig 3:**
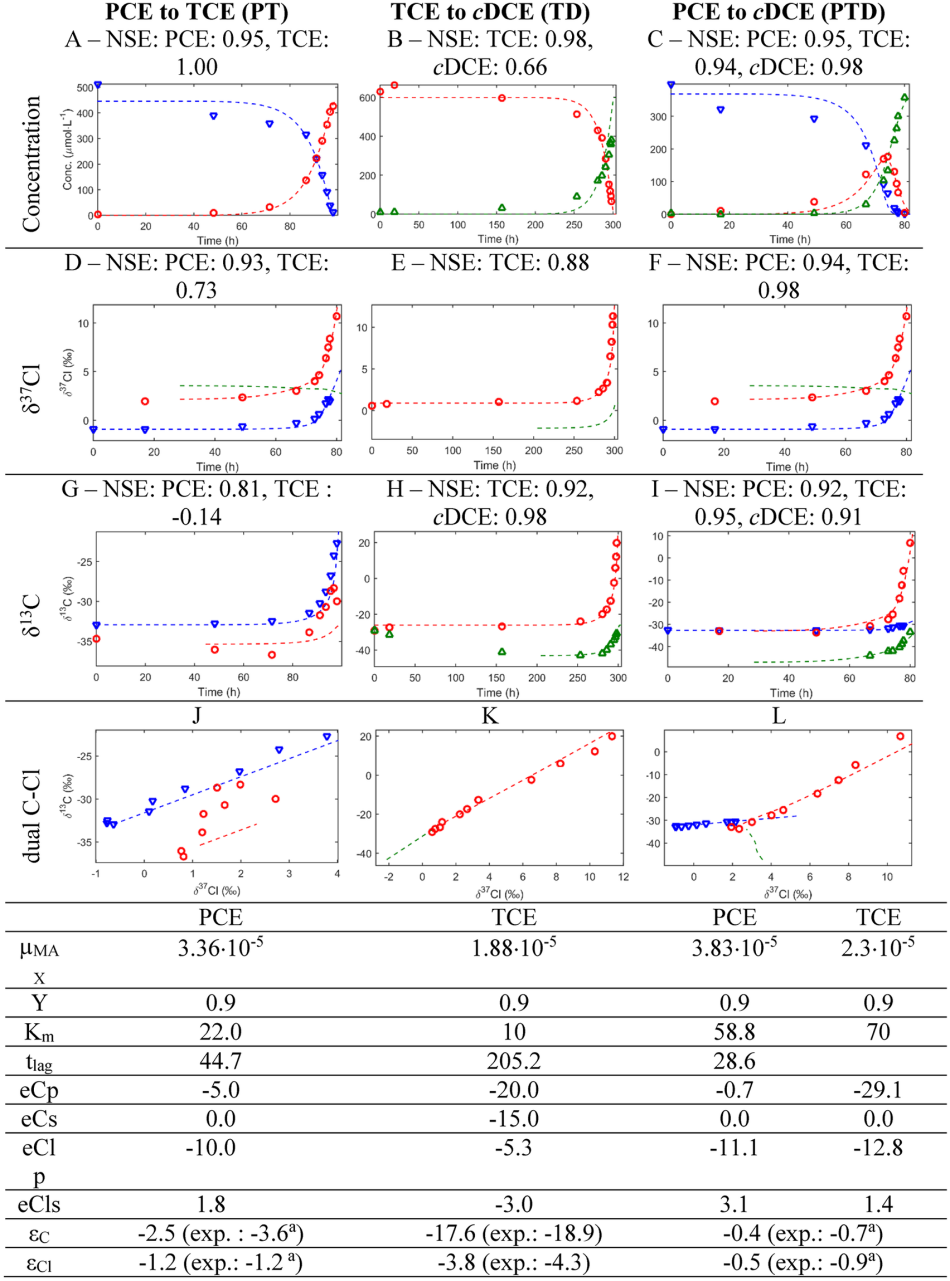
Simulation results, experimental results (one replicate per experiment), and corresponding optimized parameters. NSE: Nash Sutcliff Coefficient. Blue, green and red lines correspond to simulated PCE, TCE, and *c*DCE, respectively. Red circles, blue triangles and green triangles correspond to experimental PCE, TCE and *c*DCE. μ_MAX_ is given in μmol·g of protein^-1^·s^-1^; Y is given in g of protein·(μmol of released chloride)^-1^; K_m_ is given in μmol·L^-1^; isotopic effects e are given in ‰; t_lag_ is given in h; P, T, D stand for PCE, TCE and *c*DCE; p and s stand for primary and secondary. ε_C_ and ε_Cl_ are the overall C and Cl enrichment factors in ‰ determined by application of Rayleigh to the simulated data. Corresponding ε_C_ and ε_Cl_ experimentally determined are given in brackets. ^a^: Badin et al. [27].

The kinetic parameters (i.e., μ_MAX_ and K_m_) chosen for all simulations were comparable to those shared by Yu and Semprini [24] and Maymo-Gatell et al. [25] while the selected C and Cl enrichment factors are in the generally observed experimental range [22, 26] (Fig 3 and Table F in S1 File). Simulations were performed until the concentration of the degraded compound reached 1% of the initial concentration, thus remaining in an experimentally representative context.

### Comparison method

In order to compare both models and to determine in which cases the detail of the GM is necessary, 31 different sets of isotopic parameters were defined: some which differed in C and Cl enrichment factors, some with no secondary isotopic effect, and some with normal/inverse secondary isotopic effects. Simulations were then performed for each set of parameters with both models (GM and SM). The average isotopic effect of the three Cl atoms located in non-reacting positions was used as Cl secondary isotopic effect in SM. These isotopic parameters are summarised in Table A in S1 File.

In order to evaluate the goodness of fit between SM and GM with regards to chloroethenes concentration and isotopic behaviour along degradation, the Nash-Sutcliff efficiency coefficient (NSE) [28] and a normalised maximal absolute error coefficient (NME) were determined. NSE varies between—∝ and 1, a value of 1 corresponding to a perfect fit and is given by:

$$NSE=1-\frac{\sum_{i=1}^{N}{\left(S_{i}-Q_{i}\right)}^{2}}{\sum_{i=1}^{N}{\left(Q_{i}-Q\right)}^{2}}$$ (11)

The NME is given in percent and corresponds to the maximal absolute difference between GM and SM, divided by the maximal amplitude of GM. Compared to the established NSE coefficient, the NME allows a more conservative comparison between the two models and represents the worst case error. The discrepancy ratio η previously defined by Jin et al. [13] was additionally used to evaluate the bias in SM due to the fact that we are solving the differential equations for C and Cl isotopologues of a compound independently and thus twice simulating this compound. The more the value of η deviates from 1, the higher is the discrepancy between compound concentrations simulated with the C and Cl systems. NSE, NME and η determined for all parameter sets are given in Tables B and C in S1 File.

### Results of GM vs. SM comparison

NSEs of 0.995 to 1 were obtained for all simulated species (i.e. biomass, chloroethenes concentrations and isotopic ratios) when comparing GM and SM simulated data using the same set of parameters. This indicates that both models simulate chloroethenes concentrations and isotopic behaviours almost identically. An exception is observed in the case of *c*DCE Cl isotopic composition where some NSEs ranging from -3 to -16 are obtained. Such deviation however occurs only when non-reactive Cl atoms in PCE show different secondary isotope effects depending on their position relative to the reacting bond (PTD_diff_sec, Table B in S1 File). The observed discrepancy results from the unequal Cl isotope distribution in TCE due to the occurrence of different secondary Cl isotopic effects during transformation of PCE to TCE. The fact that this degradation step further affects the *c*DCE Cl isotopic composition is taken into account when applying the GM but is lost when applying the SM, hence explaining the discrepancy. This *c*DCE specific discrepancy is reflected by NME ranging from 47 to 94% as well (Table C in S1 File). However, the absolute maximum difference in *c*DCE Cl isotopic composition between GM and SM is actually of 2 ‰ which is only slightly higher than the analytical uncertainty. Such discrepancy thus poses a problem primarily when little overall shifts are observed in *c*DCE Cl isotopic composition. Apart from this special case, a maximum NME of 2% was found associated with *c*DCE C isotopic composition during TCE degradation to *c*DCE where inverse secondary isotopic effects were considered. Among the 31 simulations with different parameter sets, NME was < 1% in 96% of the cases (Table C in S1 File). These results confirm that GM and SM both simulate chloroethenes concentrations and isotopic behaviours almost identically except for *c*DCE Cl isotopic composition when different secondary isotopic effects occur during the transformation step of PCE to TCE in the overall degradation of PCE to *c*DCE. Finally, acceptable discrepancy values η related to the simultaneous resolution of differential equation systems for compounds relatively to C and Cl ranging from 0.979 to 1.008 were observed.

As the discrepancy between SM and GM is negligible in most cases and as η remains in a reasonable range for the 31 sets of simulations, SM can be considered as a sufficiently representative model for most cases. *c*DCE Cl isotopic composition from PCE to *c*DCE degradation where different secondary isotopic effects are considered during the transformation step of PCE to TCE constitutes the only exception. While previous studies have calculated different position-specific isotope effects for some compounds [29], these effects have not been experimentally observed for chlorinated ethenes. Thus, it is not presently clear whether the phenomenon investigated in this exceptional case, which would necessitate the GM, occurs in reality. Finally, depending on the required simulation accuracy, either GM or SM may be chosen.

### Model responses

Graphs representing the chloroethenes concentrations, C and Cl evolution as a function of time as well as dual C-Cl isotope plots obtained for a selection of the 31 simulation sets are given in Table D in S1 File. All simulations responded as expected for the different sets of isotopic effects (Fig 3 and Table D in S1 File). For example, for one element, no enrichment was observed when all enrichments associated with this element were set to 0 ‰. When secondary effects were set to 0 for Cl, the initial Cl isotopic composition of TCE equalled that of PCE for PT (e.g. PT_no_sec, Table D in S1 File). On the contrary, when a normal secondary isotopic effect (i.e., ε_ClSec_ < 0 ‰) was set for Cl, the initial Cl isotopic composition of TCE was lighter than that of PCE for PT (e.g. PT_normal_sec, Table D in S1 File).

## Experimental data simulation by model SM

### Method

We have shown that when evaluating and comparing GM and SM both models almost identically simulate chloroethenes concentrations and the evolution of their relative C and Cl isotopic compositions along reductive dechlorination of PCE to *c*DCE within an experimentally plausible frame. More particularly, the goodness of fit between SM and GM is confirmed by NME < 1% and the applicability of SM is supported by η > 0.989 for the sets of parameters used to fit the experimental data. Simulations performed to assess to what extent the developed model can truly reproduce experimental data were hence carried out with SM. Results from a former study, as well as additional experiments whose methods and results are described in S1 File, were simulated. Simulations were fit on one replicate of each set of experiments, i.e. for one replicate of reductive dechlorination of PCE to TCE (PT), PCE to *c*DCE (PTD), and TCE to *c*DCE (TD), respectively. Experimentally observed lag phases *t*_lag_ of 45 h, 29 h and 205 h for PT, PTD, and TD, respectively, were taken into account when simulating the experimental data. Simulations were performed for periods corresponding to the experimentally observed degradation time.

The kinetic parameters (μ_MAX_, *K*_*m*_) and *t*_lag_ were optimized first, followed by optimization of isotopic parameters (*ε*). The resulting kinetic parameters are in the same range as previously reported values [22, 23]. Isotopic parameters were optimized based on a range varying around experimentally determined C enrichment factor and Cl primary and secondary isotopic effects. The goodness of fit between simulated and experimental data was evaluated by NSE.

### Results

Chloroethene concentrations and isotopic composition plots of both simulated and experimental data are shown in Fig 3, as are NSEs and optimized model parameters. Concentrations simulated with Monod kinetics are in strong agreement with experimentally measured concentrations (NSEs from 0.66 to 1.00). Isotopic compositions also show generally good agreement with NSEs > 0.91 in 67% of cases. Notably, the unusual inverse secondary Cl isotopic effect observed for PT and PTD when assuming a one-step scenario could be simulated. This indicates that the developed modelling approach can reliably predict experimental data (Fig 3). One clear outlier is the C isotopic composition of TCE associated with PT where a NSE of -0.14 was determined where a poorer agreement between simulated and experimental data is observed. This is consistent with the large variability of observed dual C-Cl isotope slopes associated with TCE from PT between experimental replicates which hindered determination of a unique dual C-Cl isotope slope associated with TCE from PT (Fig C and Table E in S1 File).

## Conclusions

The dynamics of C and Cl isotopes of PCE, TCE and *c*DCE during sequential reductive dechlorination when taking into account secondary isotope effects and Monod kinetics were successfully simulated when C and Cl isotopes were considered both simultaneously (GM) and separately (SM). Except for a specific case, NSEs > 0.995 and NMEs < 2% were obtained when comparing GM and SM simulated data. This indicates that both models almost identically simulate chloroethenes reductive dechlorination for a large set of isotopic effect combinations. The only exception applies for the *c*DCE Cl isotopic composition when differential Cl secondary isotopic effects are considered for the PCE to TCE dechlorination step during PCE to *c*DCE degradation. Here, only GM is able to produce reliable results. However, this type of phenomenon has not yet been documented experimentally for chlorinated hydrocarbons. While GM represents the most accurate and detailed way to simulate the evolution of isotopic composition over time during degradation, the less complex and computationally demanding model SM has been shown to be applicable for a majority of cases. Simulation of experimental data was performed with the models for PCE dechlorination to TCE (PT) and *c*DCE (PTD) as well as for TCE dechlorination to *c*DCE (TD). All models faithfully reproduced the experimental data with NSE > 0.66 except for TCE C isotopic composition where NSE = -0.14, reflecting the inconsistency between experimental replicates. These results underscore the potential for further incorporation of isotope data into reactive transport models simulating processes occurring at multiple scales.

Finally, as highlighted by Meckenstock et al. [30], there is a need for understanding processes affecting contaminant degradation at various scales (from conceptual model of aquifers to mass transfer through cell membranes and biochemical enzymatic reaction) in order to increase the accuracy of plume fate prediction where biodegradation occurs. These results offer the possibility to integrate information on processes occurring at the organism scale into models addressing contaminant degradation at the aquifer scale.

## Supporting information

S1 File Supporting information to the article. Additional schematic explanations, initial isotopologue/isotopocule concentration calculation, final isotopic composition calculation, tables summarising parameters used for the simulations as well as a selection of simulation results and corresponding NSE and NME, experimental methods and results, and differential equations used in COMSOL.
(PDF)

S2 File Zip file containing documented Matlab and COMSOL files. The COMSOL and Matlab files used for the modelling aspects of the paper. A detailed “read me” document describing the individual Matlab files is also supplied.
(ZIP)
