# Supplementary material for: Modelling of C/Cl isotopic behaviour during chloroethene biotic reductive dechlorination: Capabilities and limitations of simplified and comprehensive models
Source: PLoS One. 2018 Aug 22;13(8):e0202416. doi: 10.1371/journal.pone.0202416 (PMC6104987; doi:10.1371/journal.pone.0202416)
Supplement: S1 File — Additional schematic explanations, initial isotopologue/isotopocule concentration calculation, final isotopic composition calculation, tables summarising parameters used for the simulations as well as a selection of simulation results and corresponding NSE and NME, experimental methods and results, and differential equations used in COMSOL. (PDF) [file pone.0202416.s001.pdf]

Supporting Information to:

Modelling of C/Cl isotopic behaviour during chloroethene biotic reductive dechlorination: capabilities and limitations of simplified and comprehensive models

doi:*10.1371/journal.pone.0202416*

Alice Badin[1], Fabian Braun[2,3], Landon J.S. Halloran[1], Julien Maillard[4], Daniel Hunkeler[1]

[1]University of Neuchâtel, Centre for Hydrogeology & Geothermics (CHYN), Rue Emile Argand 11, CH-2000 Neuchâtel, Switzerland

[2]Swiss Center for Electronics and Microtechnology (CSEM), Systems Division, Rue Jaquet-Droz 1, CH-2002 Neuchâtel, Switzerland

[3]Ecole Polytechnique Fédérale de Lausanne (EPFL), Signal Processing Laboratory (LTS5), Station 11, CH-1015 Lausanne, Switzerland

[4]Ecole Polytechnique Fédérale de Lausanne (EPFL), Laboratory for Environmental Biotechnology, Station 6, CH-1015 Lausanne, Switzerland

1. Mathematical model development

1.1. Separate consideration of C and Cl isotopes (SM)

Here, a schematic explanation of the transitions considered between isotopologues of PCE, TCE and *c*DCE during PCE reductive dechlorination for the simplified model (SM) is given (Figure A). The matrices of isotopologue/isotopocule fractionation factors used in SM associated with each step of reductive dechlorination of PCE to *c*DCE are also given (Figure B).

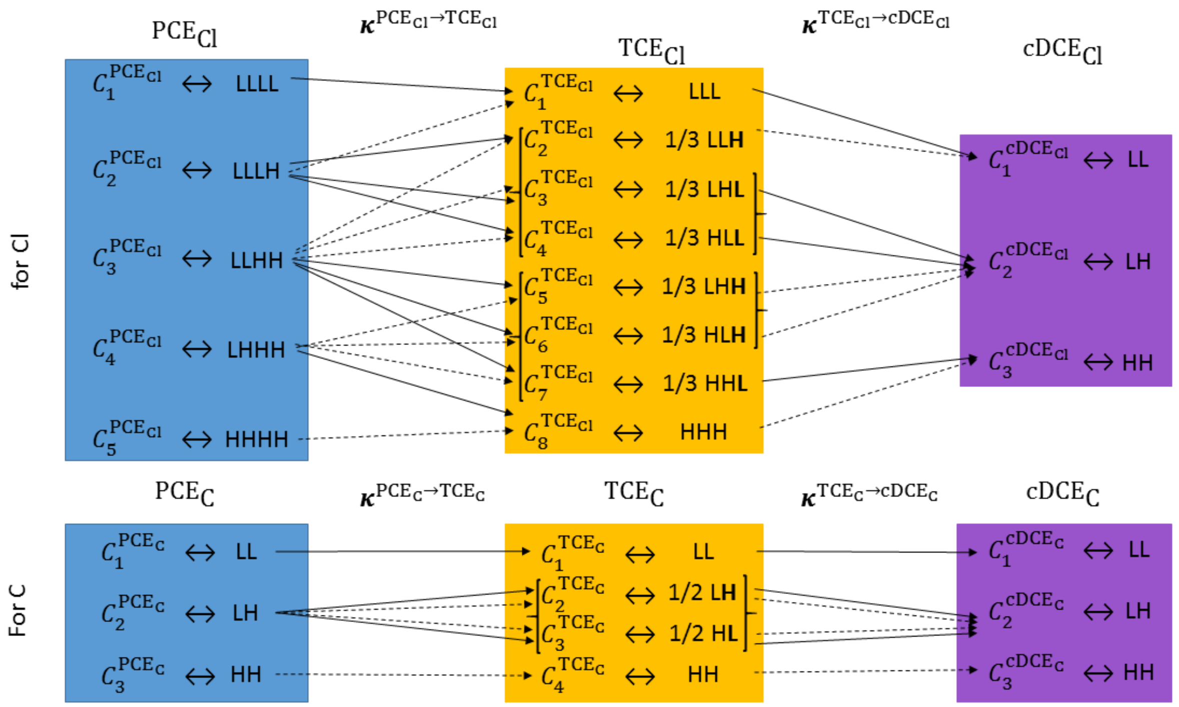

Figure A Schematic explanation of the transitions considered between isotopologues of PCE, TCE and *c*DCE during PCE reductive dechlorination for the simplified model (SM). A more intuitive notation was used in this scheme based on the scheme suggested by Hunkeler et al. [11]: L designates light isotopes while H designates heavy isotopes (of either C or Cl). Solid arrows correspond to transitions where bond breakage involves a light isotope of the considered element while dotted arrows correspond to transitions involving heavy isotopes. $\boldsymbol{\kappa}$ are the matrices containing the isotopologue fractionation factors relative to Cl and C associated with each transition. The corresponding matrices are given in Figure B.

$$
\boldsymbol{\kappa}^{\mathrm{PCE}_C\rightarrow\mathrm{TCE}_C}=
\begin{array}{c}
\begin{array}{cccc} \mathrm{LL} & \mathrm{LH} & \mathrm{HL} & \mathrm{HH} \end{array}\\
\begin{pmatrix}
1 & 0 & 0 & 0\\
0 & \frac{1}{4\,A_p^C}+\frac{1}{4\,A_s^C} & \frac{1}{4\,A_p^C}+\frac{1}{4\,A_s^C} & 0\\
0 & 0 & 0 & \frac{1}{A_p^C\,A_s^C}
\end{pmatrix}
\end{array}
\begin{array}{l}\mathrm{LL}\\ \mathrm{LH}\\ \mathrm{HH}\end{array}
$$

$$
\boldsymbol{\kappa}^{\mathrm{PCE}_{Cl}\rightarrow\mathrm{TCE}_{Cl}}=
\begin{array}{c}
\begin{array}{cccccccc} \mathrm{LLL} & \mathrm{LLH} & \mathrm{LHL} & \mathrm{HLL} & \mathrm{LHH} & \mathrm{HLH} & \mathrm{HHL} & \mathrm{HHH} \end{array}\\
\begin{pmatrix}
1 & 0 & 0 & 0 & 0 & 0 & 0 & 0\\
\frac{1}{4\,A_p^{Cl}} & \frac{1}{4\,A_s^{Cl}} & \frac{1}{4\,A_s^{Cl}} & \frac{1}{4\,A_s^{Cl}} & 0 & 0 & 0 & 0\\
0 & \frac{1}{6\,A_p^{Cl}\,A_s^{Cl}} & \frac{1}{6\,A_p^{Cl}\,A_s^{Cl}} & \frac{1}{6\,A_p^{Cl}\,A_s^{Cl}} & \frac{1}{6\,(A_s^{Cl})^2} & \frac{1}{6\,(A_s^{Cl})^2} & \frac{1}{6\,(A_s^{Cl})^2} & 0\\
0 & 0 & 0 & 0 & \frac{1}{4\,A_p^{Cl}\,(A_s^{Cl})^2} & \frac{1}{4\,A_p^{Cl}\,(A_s^{Cl})^2} & \frac{1}{4\,A_p^{Cl}\,(A_s^{Cl})^2} & \frac{1}{4\,(A_s^{Cl})^3}\\
0 & 0 & 0 & 0 & 0 & 0 & 0 & \frac{1}{A_p^{Cl}\,(A_s^{Cl})^3}
\end{pmatrix}
\end{array}
\begin{array}{l}\mathrm{LLLL}\\ \mathrm{LLLH}\\ \mathrm{LLHH}\\ \mathrm{LHHH}\\ \mathrm{HHHH}\end{array}
$$

$$
\boldsymbol{\kappa}^{\mathrm{TCE}_C\rightarrow\mathrm{cDCE}_C}=
\begin{array}{c}
\begin{array}{ccc} \mathrm{LL} & \mathrm{LH} & \mathrm{HL} \end{array}\\
\begin{pmatrix}
1 & 0 & 0\\
0 & \frac{1}{A_p^C} & 0\\
0 & \frac{1}{A_s^C} & 0\\
0 & 0 & \frac{1}{A_p^C\,A_s^C}
\end{pmatrix}
\end{array}
\begin{array}{l}\mathrm{LL}\\ \mathrm{LH}\\ \mathrm{HL}\\ \mathrm{HH}\end{array}
\qquad
\boldsymbol{\kappa}^{\mathrm{TCE}_{Cl}\rightarrow\mathrm{cDCE}_{Cl}}=
\begin{array}{c}
\begin{array}{ccc} \mathrm{LL} & \mathrm{LH} & \mathrm{HL} \end{array}\\
\begin{pmatrix}
1 & 0 & 0\\
\frac{1}{A_p^{Cl}} & 0 & 0\\
0 & \frac{1}{A_s^{Cl}} & 0\\
0 & \frac{1}{A_s^{Cl}} & 0\\
0 & \frac{1}{A_p^{Cl}\,A_s^{Cl}} & 0\\
0 & \frac{1}{A_p^{Cl}\,A_s^{Cl}} & 0\\
0 & 0 & \frac{1}{(A_s^{Cl})^2}\\
0 & 0 & \frac{1}{A_p^{Cl}\,(A_s^{Cl})^2}
\end{pmatrix}
\end{array}
\begin{array}{l}\mathrm{LLL}\\ \mathrm{LLH}\\ \mathrm{LHL}\\ \mathrm{HLL}\\ \mathrm{LHH}\\ \mathrm{HLH}\\ \mathrm{HHL}\\ \mathrm{HHH}\end{array}
$$

Figure B Matrices of isotopologue/isotopocule fractionation factors used in SM associated with each step of reductive dechlorination of PCE to *c*DCE. Unlike in GM where C and Cl are considered simultaneously, C and Cl are here treated separately. Each matrix thus represents the transition between isotopologues/isotopocules of degraded to produced compound relative to either C or Cl. Letters in bold represent the reacting position. A stands for AKIE, p for primary, s for secondary. $\boldsymbol{\kappa}^{\mathrm{PCE_C}\rightarrow\mathrm{TCE_C}}$ and $\boldsymbol{\kappa}^{\mathrm{PCE_{Cl}}\rightarrow\mathrm{TCE_{Cl}}}$ thus describe the transition of PCE to TCE relative to C and Cl, respectively, while $\boldsymbol{\kappa}^{\mathrm{TCE_C}\rightarrow\mathrm{cDCE_C}}$ and $\boldsymbol{\kappa}^{\mathrm{TCE_{Cl}}\rightarrow\mathrm{cDCE_{Cl}}}$ describe the transition of TCE to *c*DCE relative to C and Cl, respectively. Symmetries (PCE and *c*DCE) and asymmetries (TCE) are also taken into account. More particularly for C where no atom is removed during the transition, it is considered during PCE transformation to TCE that primary and secondary effects are equally distributed between the two positions. On the contrary, during TCE transformation to *c*DCE, it is considered that one C either undergoes a primary or a secondary isotopic effect since the reaction is regioselective and there are therefore no equal possibilities of cleaving the bond from one or the other C.

1.2. The case of Monod kinetics: equations describing PCE, TCE and cDCE isotopocules during reductive dechlorination

For PCE reductive dechlorination to *c*DCE, the following equations apply based on equation (10), i.e. for PCE:

$$\frac{\partial C_i^{PCE}}{\partial t} = \underbrace{\left(\sum_{h=1}^{n^{\gamma-1}} \boldsymbol{\kappa}_{h,i}^{\gamma-1\rightarrow\gamma} \cdot v^{\gamma-1} \cdot \frac{C_h^{\gamma-1}}{C_{tot}^{\gamma-1}}\right)}_{\text{produced from } \gamma\text{-1}} - \underbrace{\left(\sum_{j=1}^{n^{PCE}} \boldsymbol{\kappa}_{i,j}^{PCE\rightarrow TCE}\right) \cdot v^{PCE} \cdot \frac{C_i^{PCE}}{C_{tot}^{PCE}}}_{\text{degraded to TCE}} \qquad \text{(S1)}$$

$$\forall i \in [1, \dots, n^{PCE}]$$

The first term in grey is shown (see equation (10)) but is equal to zero for PCE which is only being degraded.

For TCE :

$$\frac{\partial C_i^{TCE}}{\partial t} = \underbrace{\left(\sum_{h=1}^{n^{PCE}} \boldsymbol{\kappa}_{h,i}^{PCE\rightarrow TCE} \cdot v^{PCE} \cdot \frac{C_h^{PCE}}{C_{tot}^{PCE}}\right)}_{\text{produced from PCE}} - \underbrace{\left(\sum_{j=1}^{n^{TCE}} \boldsymbol{\kappa}_{i,j}^{TCE\rightarrow cDCE}\right) \cdot v^{TCE} \cdot \frac{C_i^{TCE}}{C_{tot}^{TCE}}}_{\text{degraded to } c\text{DCE}} \qquad \text{(S2)}$$

$$\forall i \in [1, \dots, n^{TCE}]$$

And for *c*DCE :

$$\frac{\partial C_i^{cDCE}}{\partial t} = \underbrace{\left(\sum_{h=1}^{n^{TCE}} \boldsymbol{\kappa}_{h,i}^{TCE\rightarrow cDCE} \cdot v^{TCE} \cdot \frac{C_h^{TCE}}{C_{tot}^{TCE}}\right)}_{\text{produced from TCE}} - \underbrace{\left(\sum_{j=1}^{n_i^{\gamma}} \boldsymbol{\kappa}_{i,j}^{\gamma\rightarrow\gamma+1}\right) \cdot v^{\gamma} \cdot \frac{C_i^{\gamma}}{C_{tot}^{\gamma}}}_{\text{degraded to } \gamma+1} \qquad \text{(S3)}$$

$$\forall i \in [1, \dots, n^{cDCE}]$$

The second term in grey is left here to remind of equation (10) but does not apply to *c*DCE which is only being produced.

1.3. Calculation of initial isotopocules (GM) and isotopologues (SM) concentrations

From hereon in, all equations are applicable to any of the simulated compounds γ (i.e. PCE, TCE or *c*DCE). Therefore, for the sake of readability, the γ in the superscript of the corresponding variables has been omitted. In the case of GM, the initial isotopocules concentrations can be calculated based on the initial isotopic compositions ($\delta^{37}Cl_0$ and $\delta^{13}C_0$ of degraded compound and the total initial concentration thereof). Initial isotopocules concentrations C_h can be determined from the total compound concentration C_0 based on the relative abundance of isotopocule h as formerly suggested by Jin et al. [13]:

$$C_h = C_0 \cdot p_h, \text{ with } p_h = \left(P_{^{13}\mathrm{C}}\right)^{n_{13\mathrm{C}_h}} \left(P_{^{12}\mathrm{C}}\right)^{n_{12\mathrm{C}_h}} \left(P_{^{37}\mathrm{Cl}}\right)^{n_{37\mathrm{Cl}_h}} \left(P_{^{35}Cl}\right)^{n_{35\mathrm{Cl}_h}} \quad \text{(S4)}$$

Where p_h is the relative abundance of the isotopocule h containing $n_{13\mathrm{C}_h} (= \sum_{(i \in \mathrm{C\ Atoms})} \boldsymbol{W}_{h,i})$ ^{13}C from a total of n_C $(= n_{13\mathrm{C}_h} + n_{12\mathrm{C}_h})$ C and $n_{37\mathrm{Cl}_h} (= \sum_{(i \in \mathrm{Cl\ Atoms})} \boldsymbol{W}_{h,i})$ ^{37}Cl from a total of n_Cl $(= n_{37\mathrm{Cl}_h} + n_{35\mathrm{Cl}_h})$ Cl atoms.

When C and Cl are considered separately, the relative abundance of isotopologues relative to C and Cl can be determined separately by the probability mass function, as previously described by Hunkeler, van Breukelen and Elsner [11] according to:

$$p_h = \binom{n_\mathrm{C}}{n_{13\mathrm{C}_h}} \left(P_{^{13}\mathrm{C}}\right)^{n_{13\mathrm{C}_h}} \left(P_{^{12}\mathrm{C}}\right)^{n_{12\mathrm{C}_h}}, \text{ for isotopologues relative to C} \quad \text{(S5)}$$

$$p_h = \binom{n_\mathrm{Cl}}{n_{37\mathrm{Cl}_h}} \left(P_{^{37}\mathrm{Cl}}\right)^{n_{37\mathrm{Cl}_h}} \left(P_{^{35}\mathrm{Cl}}\right)^{n_{35\mathrm{Cl}_h}}, \text{ for isotopologues relative to Cl} \quad \text{(S6)}$$

where p_h is the relative abundance of isotopologue (or isotopocule for asymmetric molecules) h. Such simplification is possible when C and Cl are considered separately since the atoms positions relative to each other are not any more taken into consideration (isotopologues) contrary to when C and Cl are considered simultaneously as in equation (S4) (isotopocules). For asymmetric chlorinated ethenes (e.g. TCE) the relative abundances are equally distributed between isotopocules arising due to asymmetry.

Isotopic ratios of an element E are usually given by the delta notation δEreported against international standards (VPDB or SMOC for C and Cl, respectively) defined as:

$$\delta E = \left(\frac{R}{R_\mathrm{std}} - 1\right) \cdot 1000\ [‰] \quad \text{(S7)}$$

where R and R_{std} are the isotope ratios of the element E of the sample and the standard, respectively (R_VPDB = 0.0111802 [31] and R_SMOC = 0.319766 [11]).

The relative abundance of C and Cl isotopes in isotopocules or isotopologues h is therefore calculated based on the initial isotopic compositions $\delta^{13}\mathrm{C}_0$ and $\delta^{37}\mathrm{Cl}_0$ as:

$$P_{^{13}\mathrm{C}} = \frac{\left(\frac{\delta^{13}\mathrm{C}_0}{1000} + 1\right) \cdot R_{\mathrm{VPDB}}}{\left(\frac{\delta^{13}\mathrm{C}_0}{1000} + 1\right) \cdot R_{\mathrm{VPDB}} + 1} \tag{S8}$$

$$P_{^{37}\mathrm{Cl}} = \frac{\left(\frac{\delta^{37}\mathrm{Cl}_0}{1000} + 1\right) \cdot R_{\mathrm{SMOC}}}{\left(\frac{\delta^{37}\mathrm{Cl}_0}{1000} + 1\right) \cdot R_{\mathrm{SMOC}} + 1} \tag{S9}$$

1.4. Final C and Cl isotopic composition

Finally, the C and Cl isotopic compositions of each compound can be computed based on the simulated isotopocule/isotopologue concentrations according to:

$$\delta^{13}\mathrm{C} = \left(\frac{R_{\mathrm{C}}}{R_{\mathrm{VPDB}}} - 1\right) \cdot 1000\ [‰],\ \text{with } R_{\mathrm{C}} = \sum_{\forall h} \left(\frac{n_{13\mathrm{C}_h}}{n_{12\mathrm{C}_h}} C_h\right) \tag{S10}$$

$$\delta^{37}\mathrm{Cl} = \left(\frac{R_{\mathrm{Cl}}}{R_{\mathrm{SMOC}}} - 1\right) \cdot 1000\ [‰],\ \text{with } R_{\mathrm{Cl}} = \sum_{\forall h} \left(\frac{n_{37\mathrm{Cl}_h}}{n_{35\mathrm{Cl}_h}} C_h\right) \tag{S11}$$

2. Models implementation, evaluation and comparison

2.1. GM vs. SM

Tables summarising sets of isotopic parameters used to perform simulations aimed at comparing GM and SM (Table A) as well as comparison results given by the NSE and NME (Tables B and C) are summarised in this section.

Table A Sets of isotopic effect parameters with which both GM and SM were run. εCα, εCβ, εClα1, εClα2, εClβ1, εClβ2 correspond to C primary, C secondary, Cl primary, Cl secondary in geminal position, Cl secondary in vicinal positions (β1 and β2), respectively. f corresponds to the fraction remaining of initial compound. No_sec, normal_sec, inv_sec, diff_sec, diff_secB, diff_secP, diff_secT are relative to secondary isotope effects and stand for no, normal, inverse, different secondary effects. Different cases are differentiated for different secondary effects associated with PTD: B means that both PCE and TCE undergo secondary effects while P and T mean that either PCE or TCE undergoes secondary effects, respectively.

	**PCE**						**TCE**						**PCE**	**TCE**
**Simulation type**	**εCα**	**εCβ**	**εClα1**	**εClα2**	**εClβ1**	**εClβ2**	**εCα**	**εCβ**	**εClα1**	**εClα2**	**εClβ1**	**εClβ2**	**f %**	**f %**
PT_1no_sec	-20	0	-0.2	0	0	0							0.9	
PT_2no_sec	-20	0	-2	0	0	0							0.9	
PT_3no_sec	-4	0	-2	0	0	0							0.9	
TD_1no_sec							-20	0	-0.2	0	0	0		0.9
TD_2no_sec							-20	0	-2	0	0	0		0.9
TD_3no_sec							-4	0	-2	0	0	0		0.9
PTD_1no_sec	-20	0	-0.2	0	0	0	-20	0	-0.2	0	0	0	1.0	
PTD_2no_sec	-20	0	-2	0	0	0	-20	0	-2	0	0	0	1.0	
PTD_3no_sec	-4	0	-2	0	0	0	-4	0	-2	0	0	0	1.0	
PT_1normal_sec	-20	-10	-10	-3	-3	-3							1.0	
PT_3normal_sec	-4	-5	-2	-3	-3	-3							0.9	
TD_1normal_sec							-20	-10	-0.2	-3	-3	-3		1.0
TD_3normal_sec							-4	-5	-2	-3	-3	-3		1.0
PTD_1normal_sec	-20	-10	-10	-3	-3	-3	-20	-10	-0.2	-3	-3	-3	0.9	
PTD_3normal_sec	-4	-5	-2	-3	-3	-3	-4	-5	-2	-3	-3	-3	1.0	
PT_1inv_sec	-20	5	-10	3	3	3							0.9	
PT_3inv_sec	-4	2	-2	3	3	3							0.9	
TD_1inv_sec							-20	5	-0.2	3	3	3		0.9
TD_3inv_sec							-4	2	-2	3	3	3		0.9
PTD_1inv_sec	-20	5	-10	3	3	3	-20	5	-0.2	3	3	3	1.0	
PTD_3inv_sec	-4	2	-2	3	3	3	-4	2	-2	3	3	3	0.9	
PT_1diff_sec	-20	3	-10	-5	2	-3							1.0	
PT_3diff_sec	-4	-3	-2	-5	2	-3							0.9	
TD_1diff_sec							-20	3	-0.2	-5	-3	2		0.9
TD_3diff_sec							-4	-3	-2	-5	-3	2		0.9
PTD_1diff_secB	-20	3	-10	-5	2	-3	-20	3	-0.2	-5	-3	2	1.0	
PTD_3diff_secB	-4	-3	-2	-5	2	-3	-4	-3	-2	-5	-3	2	1.0	
PTD_1diff_secT	-20	3	-10	-3	-3	-3	-20	3	-0.2	-5	2	2	0.9	
PTD_3diff_secT	-4	-3	-2	-3	-3	-3	-4	-3	-2	-5	2	2	1.0	
PTD_1diff_secP	-20	3	-10	-5	2	-3	-20	3	-0.2	-3	-3	-3	1.0	
PTD_3diff_secP	-4	-3	-2	-5	2	-3	-4	-3	-2	-3	-3	-3	1.0	
PT_exp	-5	0	-10	1.8	1.8	1.8							0.9	
TD_exp							-30.0	-5.0	-5.3	-3.0	-3.0	-3.0		0.8
PTD_exp	-0.7	0	-11.1	3.1	3.1	3.1	-29.1	0	-12.8	1.4	1.4	1.4	1.0	

Table B Nash-Sutcliff efficiency coefficients (NSE) associated with the comparison of GM and SM for all sets of parameters used for simulations. No NSE was calculated for cDCE Cl isotopic composition associated with TD when no secondary effect occurred since the Cl isotopic composition is then constant and equal to the initial TCE Cl isotopic composition.

**Simulation type**	**X []**	**PCE []**	**TCE []**	***c*DCE []**	**PCE $\delta^{13}C$**	**TCE $\delta^{13}C$**	***c*DCE $\delta^{13}C$**	**PCE $\delta^{37}Cl$**	**TCE $\delta^{37}Cl$**	***c*DCE $\delta^{37}Cl$**
PT_1no_sec	1.00	1.00	1.00		1.00	1.00		1.00	1.00	
PT_2no_sec	1.00	1.00	1.00		1.00	1.00		1.00	1.00	
PT_3no_sec	1.00	1.00	1.00		1.00	1.00		1.00	1.00	
TD_1no_sec	1.00		1.00	1.00		1.00	1.00		1.00	X
TD_2no_sec	1.00		1.00	1.00		1.00	1.00		1.00	X
TD_3no_sec	1.00		1.00	1.00		1.00	1.00		1.00	X
PTD_1no_sec	1.00	1.00	1.00	1.00	1.00	1.00	1.00	1.00	1.00	1.00
PTD_2no_sec	1.00	1.00	1.00	1.00	1.00	1.00	1.00	1.00	1.00	1.00
PTD_3no_sec	1.00	1.00	1.00	1.00	1.00	1.00	1.00	1.00	1.00	1.00
PT_1normal_sec	1.00	1.00	1.00		1.00	1.00		1.00	1.00	
PT_3normal_sec	1.00	1.00	1.00		1.00	1.00		1.00	1.00	
TD_1normal_sec	1.00		1.00	1.00		1.00	1.00		1.00	1.00
TD_3normal_sec	1.00		1.00	1.00		1.00	1.00		1.00	1.00
PTD_1normal_sec	1.00	1.00	1.00	1.00	1.00	1.00	1.00	1.00	1.00	1.00
PTD_3normal_sec	1.00	1.00	1.00	1.00	1.00	1.00	1.00	1.00	1.00	1.00
PT_1inv_sec	1.00	1.00	1.00		1.00	1.00		1.00	1.00	
PT_3inv_sec	1.00	1.00	1.00		1.00	1.00		1.00	1.00	
TD_1inv_sec	1.00		1.00	1.00		1.00	1.00		1.00	1.00
TD_3inv_sec	1.00		1.00	1.00		1.00	1.00		1.00	1.00
PTD_1inv_sec	1.00	1.00	1.00	1.00	1.00	1.00	1.00	1.00	1.00	1.00
PTD_3inv_sec	1.00	1.00	1.00	1.00	1.00	1.00	1.00	1.00	1.00	1.00
PT_1diff_sec	1.00	1.00	1.00		1.00	1.00		1.00	1.00	
PT_3diff_sec	1.00	1.00	1.00		1.00	1.00		1.00	1.00	
TD_1diff_sec	1.00		1.00	1.00		1.00	1.00		1.00	1.00
TD_3diff_sec	1.00		1.00	1.00		1.00	1.00		1.00	1.00
PTD_1diff_secB	1.00	1.00	1.00	1.00	1.00	1.00	1.00	1.00	1.00	-5.42
PTD_3diff_secB	1.00	1.00	1.00	1.00	1.00	1.00	1.00	1.00	1.00	-15.83
PTD_1diff_secT	1.00	1.00	1.00	1.00	1.00	1.00	1.00	1.00	1.00	1.00
PTD_3diff_secT	1.00	1.00	1.00	1.00	1.00	1.00	1.00	1.00	1.00	1.00
PTD_1diff_secP	1.00	1.00	1.00	1.00	1.00	1.00	1.00	1.00	1.00	-3.19
PTD_3diff_secP	1.00	1.00	1.00	1.00	1.00	1.00	1.00	1.00	1.00	-7.73
PT_exp	1.00	1.00	1.00		1.00	1.00		1.00	1.00	0.00
TD_exp	1.00		1.00	1.00		1.00	1.00		1.00	1.00
PTD_exp	1.00	1.00	1.00	1.00	1.00	1.00	1.00	1.00	1.00	1.00

Table C Normalised maximum error (NME) associated with the comparison of GM and SM for all sets of parameters used for simulations, and discrepancy coefficient η reflecting the difference in evolution of compound concentrations simulated relative to C or Cl isotopologues when performing SM with all sets of parameters. No NME was calculated for *c*DCE Cl isotopic composition associated with TD when no secondary effect occurred since the Cl isotopic composition is then constant and equal to the initial TCE Cl isotopic composition.

	NME						η	
**Simulation type**	**PCE $\delta^{13}C$ %**	**TCE $\delta^{13}C$ %**	***c*DCE $\delta^{13}C$ %**	**PCE $\delta^{37}Cl$ %**	**TCE $\delta^{37}Cl$ %**	***c*DCE $\delta^{37}Cl$ %**	**PCE**	**TCE**
PT_1no_sec	0.1	0.0		0.1	0.0		1.001	
PT_2no_sec	0.1	0.0		0.1	0.0		0.999	
PT_3no_sec	0.1	0.0		0.0	0.0		0.998	
TD_1no_sec		0.2	0.0		0.2	X		1.001
TD_2no_sec		0.2	0.0		0.2	X		0.999
TD_3no_sec		0.1	0.0		0.0	X		0.998
PTD_1no_sec	0.0	0.4	0.8	0.1	0.0	0.0	1.001	
PTD_2no_sec	0.1	0.3	0.8	0.1	0.0	0.0	0.999	
PTD_3no_sec	0.1	0.0	0.2	0.0	0.0	0.0	0.998	
PT_1normal_sec	0.6	0.2		0.1	0.0		0.980	
PT_3normal_sec	0.3	0.1		0.0	0.0		0.988	
TD_1normal_sec		0.4	0.1		0.3	0.1		0.995
TD_3normal_sec		0.3	0.1		0.1	0.0		0.991
PTD_1normal_sec	0.5	0.2	0.3	0.1	0.0	0.0	0.980	
PTD_3normal_sec	0.3	0.2	0.2	0.0	0.0	0.0	0.988	
PT_1inv_sec	0.1	0.0		0.1	0.0		1.000	
PT_3inv_sec	0.2	0.1		0.0	0.0		1.008	
TD_1inv_sec		0.0	0.1		0.1	0.0		1.008
TD_3inv_sec		0.1	0.0		0.0	0.0		1.005
PTD_1inv_sec	0.0	0.9	1.7	0.0	0.0	0.0	1.000	
PTD_3inv_sec	0.2	0.4	0.7	0.0	0.0	0.0	1.008	
PT_1diff_sec	0.5	0.2		0.0	0.1		0.983	
PT_3diff_sec	0.2	0.1		0.1	0.1		0.991	
TD_1diff_sec		0.2	0.1		0.9	0.3		0.997
TD_3diff_sec		0.2	0.1		0.6	0.3		0.995
PTD_1diff_secB	0.4	0.4	1.3	0.0	0.0	56.7	0.983	
PTD_3diff_secB	0.2	0.1	0.1	0.1	0.1	93.8	0.991	
PTD_1diff_secT	0.5	0.4	1.3	0.0	0.0	0.1	0.979	
PTD_3diff_secT	0.3	0.2	0.1	0.0	0.1	0.1	0.988	
PTD_1diff_secP	0.4	0.4	1.3	0.0	0.0	46.9	0.983	
PTD_3diff_secP	0.2	0.2	0.1	0.1	0.0	69.8	0.991	
PT_exp	0.1	0.1		0.0	0.0		0.995	
TD_exp		0.6	0.2		0.34	0.07		0.989
PTD_exp	0.0	0.2	0.2	0.0	0.0	0.1	0.998	

2.2. <u>Model responses</u>

Plots representing the evolution of C and Cl isotopic compositions with time for each type of simulated isotopic effect as well as corresponding C-Cl isotope slopes are given in this section.

Table D Plots representing the evolution of C and Cl isotopic compositions with time for each type of simulated isotopic effect as well as corresponding C-Cl isotope slopes. PCE, TCE and *c*DCE are represented in blue, green and red, respectively. Plots are given for simulation with GM.

simulation type	$\delta^{13}C$	$\delta^{37}Cl$	dual C-Cl isotope slopes
PT_1no_sec	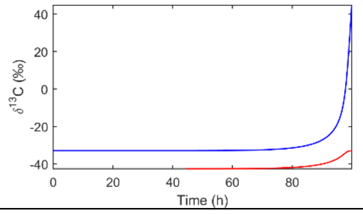	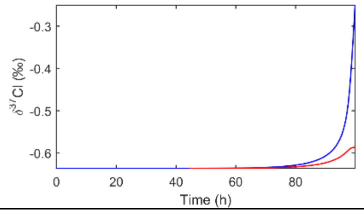	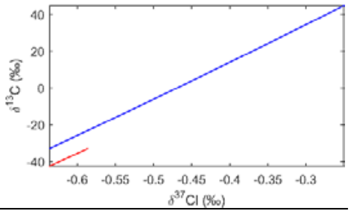
TD_1no_sec	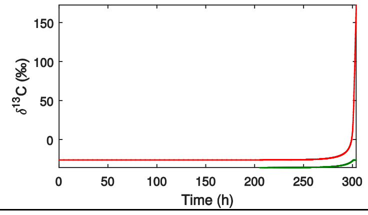	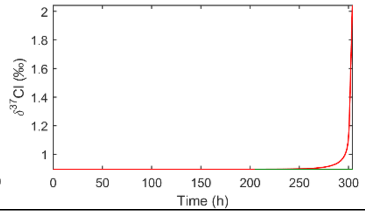	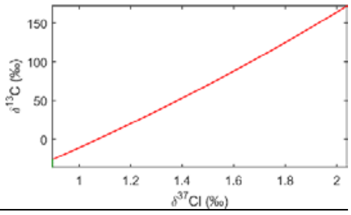
PTD_1no_sec	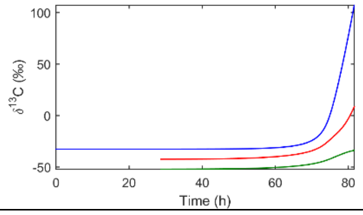	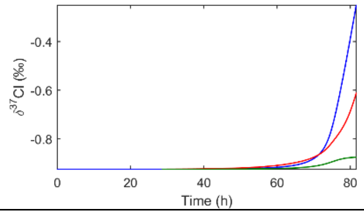	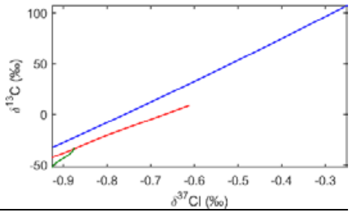
PT_1normal_sec	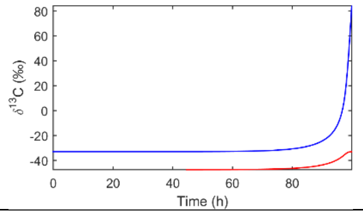	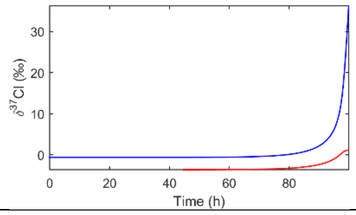	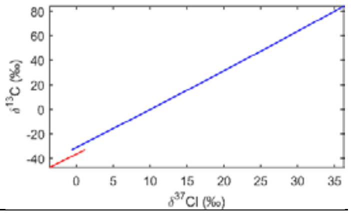
TD_1normal_sec	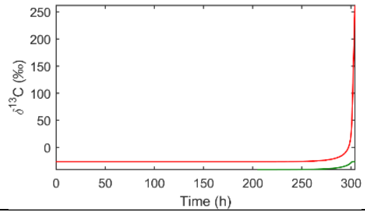	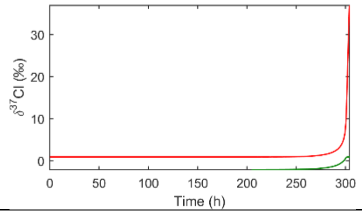	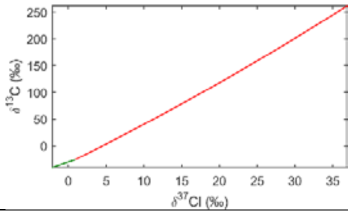
PTD_1normal_sec	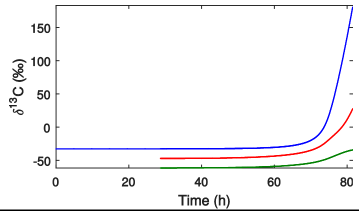	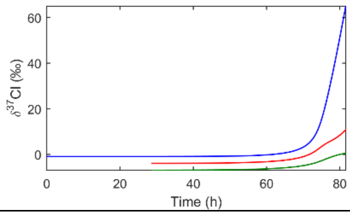	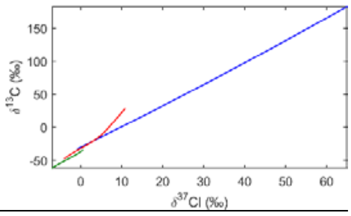

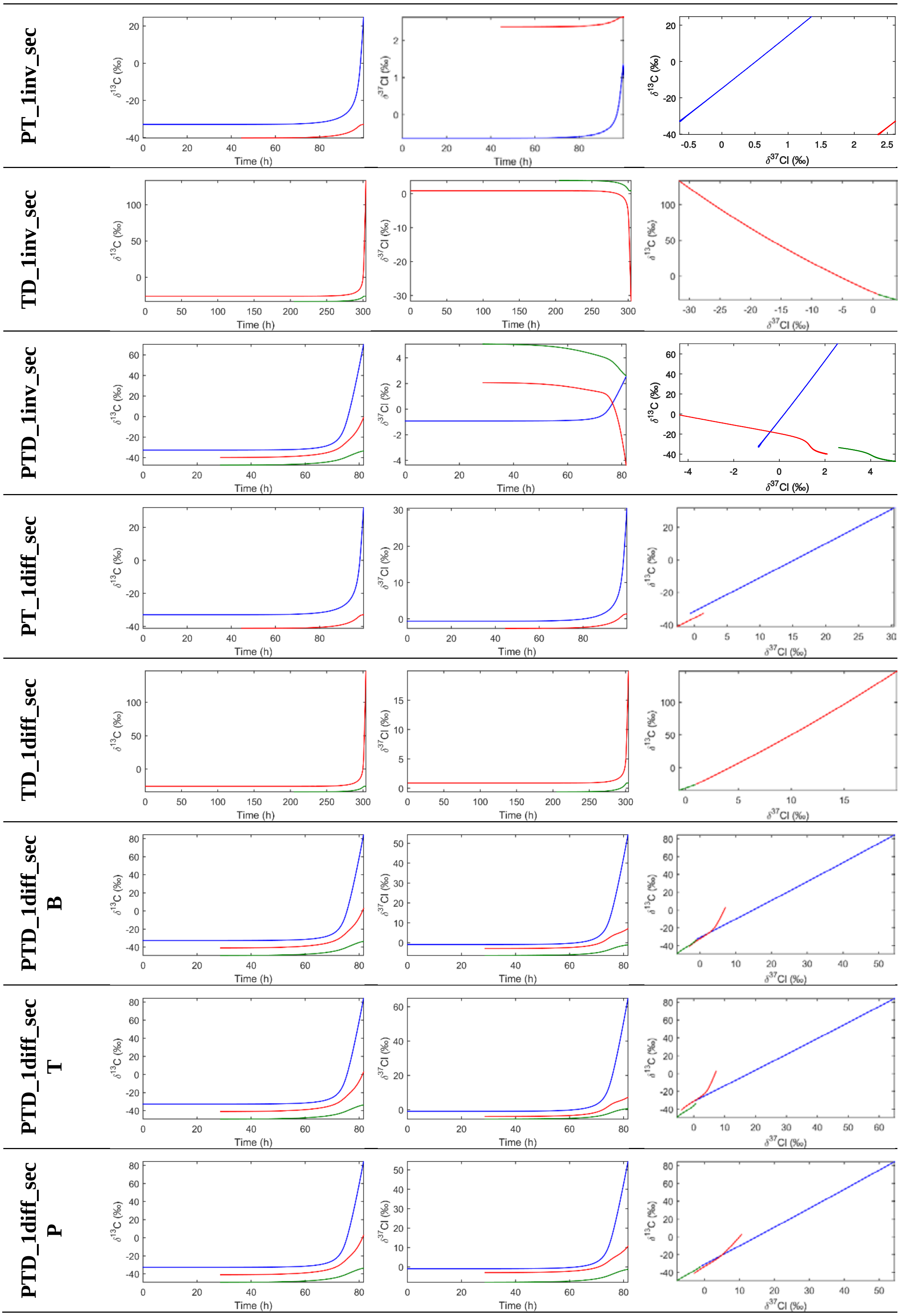
PT_1inv_sec
TD_1inv_sec
PTD_1inv_sec
PT_1diff_sec
TD_1diff_sec
PTD_1diff_sec B
PTD_1diff_sec T
PTD_1diff_sec P
δ13C (‰)
δ37Cl (‰)
Time (h)

3. Experimental work

3.1. Cultivation of bacterial consortia and sampling

PCE C and Cl isotopic data from two sets of experiments previously described[26] where reductive dechlorination of chlorinated ethenes was carried out by two bacterial consortia harbouring members of the *Sulfurospirillum* genus were used in this study. Additional C isotopic analysis of TCE and *c*DCE, and Cl isotopic analysis of TCE were performed on samples from these former experiments. Furthermore, a supplementary set of experiments was performed where reductive dechlorination of chlorinated ethenes was carried out by one of the two previously studied bacterial consortia. In summary, PCE and TCE were fed to the consortium SL2-PCEb which carried out reductive dechlorination down to *c*DCE. The corresponding reactions are referred to as PTD due to the accumulation of TCE during reductive dechlorination of PCE to *c*DCE, and TD. Reductive dechlorination of PCE by the subculture SL2-PCEc stopped at TCE (PT). The consortia cultivation and sampling was performed as described previously [27, 32]. Briefly, cultivation was carried out in 1000 mL serum bottles containing 900 mL of anaerobic medium in presence of PCE (560 µM) or TCE (560 µM) in a phosphate-bicarbonate-buffered medium where formate and acetate acted as electron donor and C source, respectively.

Isotope fractionation was characterized in three to four replicate assays per experimental set. Control experiments without addition of inoculum were also included. Aqueous samples for isotope and concentration analysis were taken to assess the remaining fractions (ranging from 100 to 5%) of initially added chlorinated ethenes. Samples were stored in glass vials sealed with PTFE-lined septa at 4 °C without headspace (for concentration analysis) or frozen upside down with headspace (for isotope analysis) after addition of NaOH 20 M to stop the dechlorination.

3.2. Analytical methods

Methods described previously were applied for concentration and isotope analysis [27]. Briefly, concentration of the aqueous samples was determined using a Thermo-Finnigan™ Trace GC Ultra gas chromatograph coupled to a Thermo-Finnigan™ DSQ II quadrupole mass spectrometry (GC-qMS).

C isotope ratios were measured using an Agilent™ 7890a gas chromatograph (GC) coupled to an Isoprime™ 100 isotope ratio mass spectrometer (IRMS) via an Isoprime GC5 combustion interface and a purge-and-trap (P&T) system (Stratum, Teledyne Tekmar). Aqueous samples

were diluted prior to analysis in 40 mL glas vials with a PTFE-lined screw cap to achieve a final PCE, TCE or *c*DCE concentration of 30 µg·L^{-1}. After the diluted samples were purged with N_2 gas (40 mL·min^{-1}), the degassed compounds were retained on a Vocarb 3000 trap (VICI) ant then transferred to a cryogenic trap (Tekmar Dohrmann) connected to the GC column (DB-VRX, 60 m, 0.25 mm, 1.4 µm). The compounds were then released to the column (helium, 1.2 mL·min^{-1}) by means of a rapid temperature increase. Samples were measured in duplicate. Standard deviations σ of the in-house reference materials were 0.4 ‰ (n=47), 0.9 ‰ (n=37), and 0.3 ‰ (n=18) for PCE, TCE, and *c*DCE, respectively. The standard uncertainty of duplicate measurements was determined according to ISO guidelines [33] as σ/√2, i.e. 0.3 ‰, 0.6 ‰, and 0.2 ‰ for PCE, TCE, and *c*DCE, respectively. Samples containing reference compounds with known isotope ratios (EA-IRMS measurement) were included in each sequence to verify the method accuracy.

Cl isotope ratio analysis was performed based on gas chromatography quadrupole mass spectrometry (GC-qMS) using the method formerly developed by Aeppli et al. [3]. An Agilent 7890 GC coupled to an Agilent 5975C quadrupole mass selective detector (Santa Clara, CA, USA) was used for the analysis. Chromatographic separation was carried out by means of a DB-5 column (30 m, 0.25 mm, 0.25 µm, Agilent) with a constant helium flow of 1.2 mL·min^{-1}. In order to obtain δ values on the SMOC scale, a calibration with two external standards for both PCE and TCE was achieved, as previously recommended [4]. These standards were formerly characterized by the Holt method [34] at the University of Waterloo ($\delta^{37}Cl_{EIL1, PCE}$ = +0.3‰ and $\delta^{37}Cl_{EIL2, PCE}$ = -2.5‰, $\delta^{37}Cl_{EIL1, TCE}$ = +3.05‰ and $\delta^{37}Cl_{EIL2, TCE}$ = -2.70‰). Working PCE or TCE standards were measured every ten sample to verify the measurement stability. Samples were diluted to 100 µg·L^{-1} and analyzed five to ten times by headspace injection using a CombiPal Autosampler (CTC Analytics, Zwingen, Switzerland). The standard uncertainty is given as the standard deviation of the mean (σ/√n).

3.3. Quantification of stable isotope fractionation

The enrichment factor ε_E was determined for each experiment according to the Rayleigh equation as follows:

$$\ln \frac{\delta_t E + 1000}{\delta_0 E + 1000} = \varepsilon_E \cdot \ln f \qquad \text{(S12)}$$

where f is the reactant remaining fraction of the degraded compound at time t, E the considered element, δ_t and δ_0 are the isotope ratios of one element at time t and time 0.

Unless notified otherwise, enrichment factors and dual isotope slopes were calculated by combining $\delta^{13}C$ and $\delta^{37}Cl$ data from all replicates and results are given with a 95% confidence interval.

3.4. Experimental determination of primary and secondary isotopic effects

According to Cretnik et al. [17], primary and secondary isotopic effects during PCE biotic reductive dechlorination can be estimated based on the difference in Cl isotopic composition between the substrate PCE and the produced TCE and Cl^-. This is based on the assumption that the products mirror the Cl isotope enrichment behaviour of the four chemically equivalent positions of PCE from which they origin. It was more specifically demonstrated that:

$$\delta^{37}\mathrm{Cl}_{\mathrm{TCE},t} = \delta^{37}\mathrm{Cl}_{\mathrm{PCE,0}} - \frac{1}{4}\varepsilon_{\mathrm{diff}} - \varepsilon_{\mathrm{PCE}} \cdot \frac{f \cdot \ln(f)}{1-f} \quad \text{(S13)}$$

After determining the Cl enrichment factor ε_{PCE} associated with PT by means of the Rayleigh equation, ε_{diff} could hence be determined based on the expression above at time 0 where:

$$\lim_{f \to 1}\left(\frac{f \cdot \ln(f)}{1-f}\right) = -1 \quad \text{(S14)}$$

The primary and secondary isotopic enrichments were consecutively determined based on the following expressions, assuming a one-step dechlorination scenario where no intermediate specie exists between PCE and TCE:

$$\varepsilon_{\mathrm{PCE\ prim}} = \delta^{37}\mathrm{Cl}_{\mathrm{Cl-,0}} - \delta^{37}\mathrm{Cl}_{\mathrm{PCE,0}} = \frac{3}{4}\varepsilon_{\mathrm{diff}} + \varepsilon_{\mathrm{PCE}} \quad \text{(S15)}$$

$$\varepsilon_{\mathrm{PCE\ sec}} = \delta^{37}Cl_{\mathrm{TCE,0}} - \delta^{37}Cl_{\mathrm{PCE,0}} = -\frac{1}{4}\varepsilon_{\mathrm{diff}} + \varepsilon_{\mathrm{PCE}} \quad \text{(S16)}$$

where $\varepsilon_{\mathrm{PCE\ prim}}$ and $\varepsilon_{\mathrm{PCE\ sec}}$ correspond to the primary and secondary isotopic effects associated with PT, respectively.

$\varepsilon_{\mathrm{PCE\ prim}}$, $\varepsilon_{\mathrm{PCE\ sec}}$, and $\varepsilon_{\mathrm{diff}}$ are given as the average between values obtained for each replicate and the uncertainties was determined by error propagation.

Since $\delta^{37}Cl$ of *c*DCE could not be measured, no information regarding secondary and primary isotopic effects during PTD or TD could be determined.

3.5. Experimental results

Whether PCE was dechlorinated by consortium SL2-PCEb or SL2-PCEc, the lag phase lasted between 20 and 50 h while the lag phase preceding TCE dechlorination as a substrate lasted one to two orders of magnitude longer, i.e. from 200 to 1500 h depending on the replicate. Detailed experimental results with regards to isotopic behaviour can be found in Figure C and Table E.

As reported previously [27], PCE was slightly enriched in heavy C and Cl during its dechlorination, with maximum enrichment factors of -3.6 ± 0.2‰ for C and -1.2 ± 0.1‰ for Cl associated with consortium SL2-PCEc (Table E). Conversely, both C and Cl enrichment were stronger for TCE dechlorination (only possible by consortium SL2-PCEb) where a C enrichment factor of -18.9 ± 1.3‰ and a Cl enrichment factor of -4.3 ± 0.4‰ were determined (Table E). While C enrichment factors of PCE are in the lower range of the so far available values [22] both C and Cl enrichment factors associated with TCE dechlorination are higher than the maximum values available of -18.5‰ for C [35] and -3.6‰ for Cl [20, 21]

The TCE dual C-Cl isotope slopes of 4.3 ± 0.2 and 3.7 ± 0.4 associated with TD and PTD, respectively, fall in the lower range corresponding to dual C-Cl isotope slopes associated with TCE microbial dechlorination (3.4 ± 0.2 [20] and 4.8 [21]; Table E and Figure C). No clear C-Cl dual isotope slope could be determined for TCE from PT by SL2-PCEc. Slopes ranging from 2.1 ± 1.1 to 3.8 ± 3.2 with a R^2 varying from 0.59 to 0.89 was determined for each replicate (Table E and Figure C), yet a dual C-Cl slope determined based on the combination of data from all replicates has no significance as its associated R^2 is of 0.16. On the contrary, the TCE dual C-Cl slope obtained for PTD by SL2-PCEb shows a high value of R^2 = 0.93 (Table E and Figure C).

Although primary and secondary Cl isotope effects could not be determined for TD by SL2-PCEb due to the lack of Cl isotope data for *c*DCE, such isotope effects were determined for PT and PTD according to the method suggested by Cretnik et al. and assuming a one-step scenario (Equations (S15) and (S16)) [17]. In the case of PTD, primary and secondary isotope effects were determined similarly as for PT. The apparition of *c*DCE in very little amounts after the apparition of TCE justifies such procedure although it should be remembered that TCE further dechlorination to *c*DCE might slightly affect the determined values (Figure C, replicate C). Primary isotopic effects of -9.0 ± 0.9 ‰ and -12.8 ± 2.1 ‰ were obtained for PT and PTD, respectively (Table E). These values are slightly to twice smaller than the previously reported value of -17.0 ± 1.6 ‰ for PT by *Desulfitobacterium* sp. strain Viet1 [17]. Secondary isotopic

effects of 1.4 ± 0.2 ‰ and 3.1 ± 0.6 ‰ associated with PT and PTD, respectively, are on the other hand significantly different from the formerly reported value of -1.0 ± 0.5 ‰ [17], and reflect an unusual inverse secondary isotope effect. Such effect was previously reported during TCE abiotic reduction by zero valent iron at the laboratory scale [36] and twice in the field where *c*DCE was found to be 1 to 1.5‰ more enriched than TCE assumed to be biotically dechlorinated [37].

Table E Summary of C and Cl enrichment factors, primary and secondary Cl isotopic effects denoted as εCl,prim and εCl,sec, respectively, dual C-Cl slopes, C isotope balance and mass balance associated with reductive dechlorination of PCE to TCE (SL2-PCEc), PCE to *c*DCE (SL2-PCEb), and TCE to *c*DCE (SL2-PCEb).

Experiment	ε_{C} (‰)	ε_{Cl} (‰)	$\varepsilon_{Cl,\ prim}$ (‰)	$\varepsilon_{Cl,\ sec}$ (‰)	$\varepsilon_{Cl,\ diff}$ (‰)	dual C-Cl PCE	dual C-Cl TCE	C isotope balance	Mass balance
**PCE to TCE**	**-3.6 ± 0.2**	**-1.2 ± 0.1**	**-9.2 ± 0.9**	**1.4 ± 0.2**	**-10.6 ± 1**	**2.7 ± 0.3<br>(R^2 = 0.94, n=23)**			
**PCE to TCE A**	-3.9 ± 0.1	-1.1 ± 0.2	-8.2 ± 0.4	1.3 ± 0.2	-9.6 ± 0.4		2.7 ± 0.8<br>(R^2 = 0.89, n=9)	-32.9 to -31.0	60%
**PCE to TCE B**	-3.7 ± 0.4	-1.5 ± 0.3	-10.1 ± 0.6	1.4 ± 0.3	-11.5 ± 0.6		3.8 ± 3.2<br>(R^2 = 0.59, n=8)	-32.9 to -29.8	90%
**PCE to TCE C**	-3.3 ± 0.7	-1.2 ± 0.2	-9.8 ± 0.4	1.6 ± 0.2	-11.5 ± 0.4		2.1 ± 1.1<br>(R^2 = 0.81, n=9)	-33.4 to -33.5	90%
**PCE to TCE D**	-3.4 ± 0.3	-1.2 ± 0.1	-8.7 ± 0.4	1.3 ± 0.3	-10 ± 0.4		3.4 ± 1.3<br>(R^2 = 0.74, n=11)	-33.2 to -32.6	90%
**PCE to *c*DCE**	**-0.7 ± 0.1**	**-0.9 ± 0.1**	**-12.8 ± 2.1**	**3.1 ± 0.6**	**-15.9 ± 2.8**	**0.7 ± 0.2<br>(R^2 = 0.74, n=29)**	**3.7 ± 0.4<br>(R^2 = 0.93, n=30)**		
**PCE to *c*DCE A**	-0.6 ± 0.2	-0.9 ± 0.1	-10.4 ± 0.3	2.3 ± 0.2	-12.8 ± 0.3			-33.1 to -33.0	85%
**PCE to *c*DCE B**	-0.7 ± 0.2	-0.8 ± 0.2	-13.3 ± 0.5	3.3 ± 0.3	-16.6 ± 0.5			-33.2 to -32.6	85%
**PCE to *c*DCE C**	-0.7 ± 0.2	-0.9 ± 0.1	-12 ± 0.3	2.9 ± 0.2	-14.9 ± 0.3			-32.4 to -32.9	80%
**PCE to *c*DCE D**	-0.8 ± 0.1	-1 ± 0.1	-15.4 ± 0.3	3.8 ± 0.2	-19.2 ± 0.3			-32.7 to -32.9	95%
**TCE to *c*DCE**	**-18.9 ± 1.3**	**-4.3 ± 0.4**					**4.3 ± 0.2<br>(R^2 = 0.99, n=29)**		
**TCE to *c*DCE A**	-20.6 ± 1.2	-4.7 ± 0.4						-29.2 to -23.6	70%
**TCE to *c*DCE B**	-17.7 ± 2.3	-3.8 ± 0.5						-27.7 to -23.3	65%
**TCE to *c*DCE C**	-18.1 ± 1.1	-4.2 ± 1						-27.8 to -23.3	60%

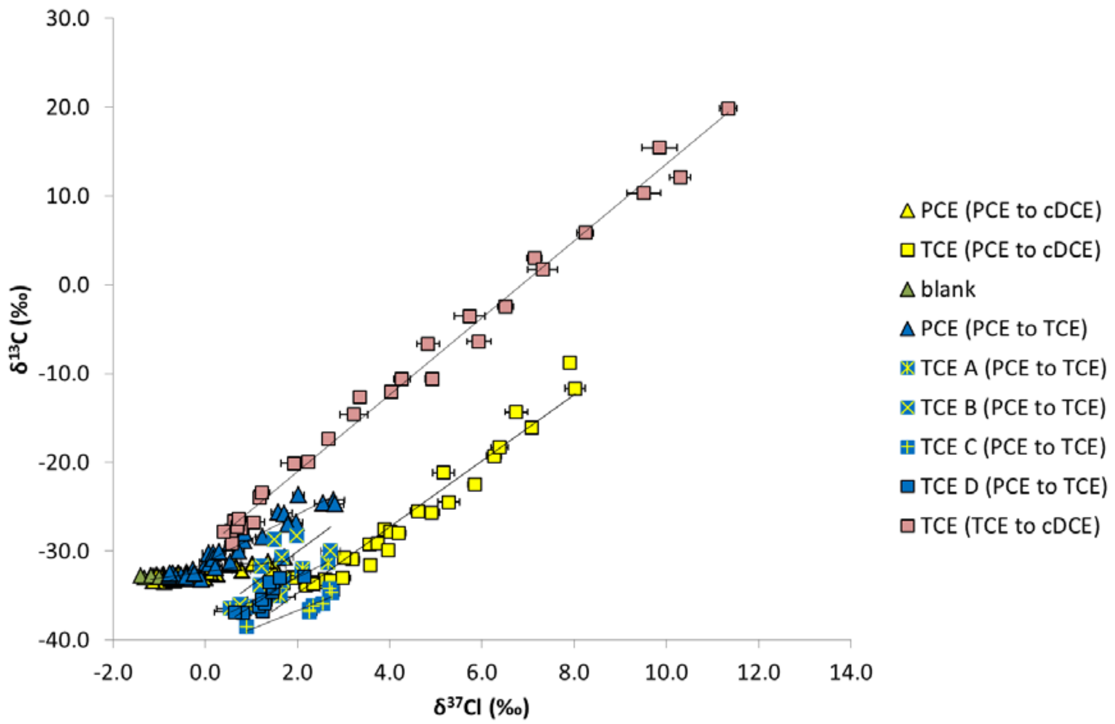

Figure C Dual C-Cl isotope slopes for PCE and TCE for reductive dechlorination of PCE to TCE (SL2-PCEc), PCE to *c*DCE (SL2-PCEb), and TCE to *c*DCE (SL2-PCEb). Except for TCE from PCE to TCE reductive dechlorination, all replicates are plotted together. For TCE from PCE to TCE reductive dechlorination, replicates A, B, C and D are plotted separately.

4. <u>Experimental data simulation by model SM</u>

The optimised parameters used for the simulations which fit the experimental data are given in this section.

Table F Summary of optimised parameters when fitting simulations to experimental data

**Parameter**	**Parameter**	**Unit**	**PT**	**TD**	**PTD**	**Description**
**MATLAB notation**	**COMSOL notation**					
**X0**	**X0**	**mg of protein.L^{-1}**	1	1	1	initial biomass concentration
**t0_delay**	**-**	**h**	45	-	29	lag phase delay
**PCE_C0**	**CP0**	**µmol.L^{-1}**	445	-	368	initial PCE concentration
**PCE_delta13C**	**d13CP0**	**‰**	-32.9	-	-32.7	initial δ^{13}C PCE
**PCE_delta37Cl**	**d37ClP0**	**‰**	-0.6	-	-0.9	initial δ^{37}Cl PCE

**TCE_C0**	**CT0**	**µmol.L^{-1}**	-	598	0	initial TCE concentration
**TCE_delta13C**	**d13CT0**	**‰**	-	-26.1		initial δ^{13}C TCE
**TCE_delta37Cl**	**d37ClT0**	**‰**	-	0.9		initial δ^{37}Cl TCE
**CDCE_C0**		**µmol.L^{-1}**	-	-	0	initial *c*DCE concentration
**CDCE_delta13C**		**‰**	-	-		initial δ^{13}C cDCE
**CDCE_delta37Cl**		**‰**	-	-		initial δ^{37}Cl cDCE
**PCE2TCE_muMax**	**uMAXP**	**µmol.mg of protein^{-1}.s^{-1}**	3.36E-05	-	3.83E-05	maximum growth rate for PCE
**PCE2TCE_Yi**	**YP**	**mg of protein.µmol of released chloride^{-1}**	0.9	-	0.9	biomass yield for PCE
**PCE2TCE_Km**	**KmP**	**µmol.L^{-1}**	22.0	-	58.8	half saturation constant for PCE
**PCE2TCE_Epsilon_Ca**	**ePCp**	**‰**	-5.0	-	-0.7	C primary effect for PCE
**PCE2TCE_Epsilon_Cb**	**ePCs**	**‰**	0.0	-	0.0	C secondary effect for PCE
**PCE2TCE_Epsilon_Cla1**	**ePClp**	**‰**	-10.0	-	-11.1	Cl primary effect /Epsilon_Clα1 for PCE
**PCE2TCE_Epsilon_Cla2**	**ePCls**	**‰**	1.8	-	3.1	Cl secondary effect /Epsilon_Clα2 for PCE
**PCE2TCE_Epsilon_Clb1**	**-**	**‰**	1.8	-	3.1	Cl secondary effect /Epsilon_Clβ1 for PCE
**PCE2TCE_Epsilon_Clb2**	**-**	**‰**	1.8	-	3.1	Cl secondary effect /Epsilon_Clβ2 for PCE
**TCE2CDCE_muMax**	**uMAXT**	**µmol.mg of protein^{-1}.s^{-1}**		1.88E-05	2.30E-05	maximum growth rate for TCE
**TCE2CDCE_Yi**	**YT**	**mg of protein.µmol of released chloride^{-1}**		0.9	0.9	biomass yield for TCE
**TCE2CDCE_Km**	**KmT**	**µmol.L^{-1}**		10.00	69.9	half saturation constant for TCE
**TCE2CDCE_Epsilon_Ca**	**eTCp**	**‰**		-30.0	-29.1	C primary effect for TCE
**TCE2CDCE_Epsilon_Cb**	**ePCs**	**‰**		-5.0	0.0	C secondary effect for TCE

**TCE2CDCE_Epsilon_Cla1**	**eTClp**	‰		-5.3	-12.8	Cl primary effect /Epsilon_Clα1 for TCE
**TCE2CDCE_Epsilon_Cla2**	**eTCls**	‰		-3.0	1.4	Cl secondary effect /Epsilon_Clα2 for TCE
**TCE2CDCE_Epsilon_Clb1**	**-**	‰		-3.0	1.4	Cl secondary effect /Epsilon_Clβ1 for TCE
**TCE2CDCE_Epsilon_Clb2**	**-**	‰		-3.0	1.4	Cl secondary effect/Epsilon_Cl β2 for TCE

References appearing only in Supporting Information:

31. Elsner, M.; Jochmann, M.; Hofstetter, T.; Hunkeler, D.; Bernstein, A.; Schmidt, T.; et al., Current challenges in compound-specific stable isotope analysis of environmental organic contaminants. *Anal. Bioanal. Chem.* **2012,** *403,* (9), 2471-2491.

32. Buttet, G. F.; Holliger, C.; Maillard, J., Functional Genotyping of Sulfurospirillum spp. in Mixed Cultures Allowed the Identification of a New Tetrachloroethene Reductive Dehalogenase. *Applied and Environmental Microbiology* **2013,** *79*, (22), 6941-6947.

33. BIPM, I., IFCC, ISO, IUPAC, IUPAP, OIML *Guide to the expression of uncertainty in measurement*; International Organization for Standardization: Geneva, Switzerland, 1993.

34. Holt, B. D.; Sturchio, N. C.; Abrajano, T. A.; Heraty, L. J., Conversion of chlorinated volatile organic compounds to carbon dioxide and methyl chloride for isotopic analysis of carbon and chlorine. *Analytical Chemistry* **1997,** *69*, (14), 2727-2733.

35. Cichocka, D.; Siegert, M.; Imfeld, G.; Andert, J.; Beck, K.; Diekert, G.; et al., Factors controlling the carbon isotope fractionation of tetra- and trichloroethene during reductive dechlorination by Sulfurospirillum ssp. and Desulfitobacterium sp. strain PCE-S. *FEMS Microbiology Ecology* **2007,** *62,* (1), 98-107.

36. Audí-Miró, C.; Cretnik, S.; Otero, N.; Palau, J.; Shouakar-Stash, O.; Soler, A.; et al., Cl and C isotope analysis to assess the effectiveness of chlorinated ethene degradation by zero-valent iron: Evidence from dual element and product isotope values. *Applied Geochemistry* **2013**, (32), 175-183.

37. Lojkasek-Lima, P.; Aravena, R.; Parker, B. L.; Cherry, J. A., Fingerprinting TCE in a Bedrock Aquifer Using Compound-Specific Isotope Analysis. *Ground Water* **2012,** *50*, (5), 754-764.
