# Supplementary material for: Modelling of C/Cl isotopic behaviour during chloroethene biotic reductive dechlorination: Capabilities and limitations of simplified and comprehensive models
Source: PLoS One. 2018 Aug 22;13(8):e0202416. doi: 10.1371/journal.pone.0202416 (PMC6104987; doi:10.1371/journal.pone.0202416)
Supplement: S2 File — The COMSOL and Matlab files used for the modelling aspects of the paper. A detailed “read me” document describing the individual Matlab files is also supplied. (ZIP) [file pone.0202416.s002.zip › Badin_et_al_PLOS_One_2018_Code_Guide.pdf]

Documentation for MATLAB and COMSOL files related to the article: **Modelling of C/Cl isotopic behaviour during chloroethene biotic reductive dechlorination: capabilities and limitations of simplified and comprehensive models**

PLOS One
*doi:*10.1371/journal.pone.0202416

Alice Badin[1], Fabian Braun[2,3], Landon J.S. Halloran[1], Julien Maillard[4], and Daniel Hunkeler[1]

[1]*University of Neuchâtel, Centre for Hydrogeology & Geothermics (CHYN), Rue Emile Argand 11, CH-2000 Neuchâtel, Switzerland*
[2]*Swiss Center for Electronics and Microtechnology (CSEM), Systems Division, Rue Jaquet-Droz 1, CH-2002 Neuchâtel, Switzerland*
[3]*Ecole Polytechnique Fédérale de Lausanne (EPFL), Signal Processing Laboratory (LTS5), Station 11, CH-1015 Lausanne, Switzerland*
[4]*Ecole Polytechnique Fédérale de Lausanne (EPFL), Laboratory for Environmental Biotechnology, Station 6, CH-1015 Lausanne, Switzerland*

August 7, 2018

Contents

1 Introduction **2**

2 Licence/Attribution **3**

3 Matlab files **3**
3.1 Files that use measured data 3
3.2 Files that model synthetic cases 4
3.3 Description of individual files 4
3.3.1 runPT.m 4
3.3.2 runTD.m 5
3.3.3 runPTD.m 5

3.3.4 readRealData.m 5
3.3.5 generateSimpleKappas.m 5
3.3.6 createParamStruct.m 6
3.3.7 tidyLatexMatrix.m 6
3.3.8 to_optimize.m 6
3.3.9 to_optimize_reduced.m 7
3.3.10 ode_isotopomere.m 7
3.3.11 plotIsotopes.m 8
3.3.12 input_data\Lab_Data.xlsx 8
3.3.13 exportData.m 8
3.3.14 exportExperiment.m 9
3.3.15 PT.mat, TD.mat & PTD.mat 9
3.3.16 brandnewkappas.mat 9
3.3.17 export_fig\ (folder) 9
3.3.18 Ghostscript 9
3.3.19 exportSynthetic.m 10
3.3.20 input_data/SimulDiffEpsilons_2Simulate.xlsx 10
3.3.21 compareMgMs.m 10
3.3.22 calcCompMetric.m 10
3.3.23 calcEpsilonRayleigh.m 11
3.3.24 nashsutcliffe.m 11

4 COMSOL Multiphysics files **12**
4.1 Overview 12
4.2 Description of individual files 12
4.2.1 PT.mph 12
4.2.2 TD.mph 12
4.2.3 PTD.mph 12
4.2.4 COMSOL_LiveLink_Template.xlsx 12

5 Credits **12**

1 Introduction

This document details the Matlab and COMSOL Multiphysics files that are used in the article:

Badin, A., Braun, F., Halloran, L.J.S., Maillard, J., & Hunkeler, D. (2018) "Modelling of C/Cl isotopic behaviour during chloroethene biotic reductive dechlorination: limitations of simplified and comprehensive models" *PLOS One*.

This work uses Matlab and COMSOL Multiphysics to implement and test two novel models: a general model (GM) and a simplified model (SM). The models describe the isotopic behavior of C and Cl isotopes during biotic reductive dechlorination of chloroethenes (PCE, TCE and *cis*-DCE) under Monod kinetics. Full details are contained in the main article and in the Supplementary info File S1.

2 Licence/Attribution

If you make use of any part of the code contained herein, the authors request that you cite the main paper by A. Badin et al. (2018) in any published works.

This program is free software: you can redistribute it and/or modify it under the terms of the GNU General Public License, version 3 as published by the Free Software Foundation. This program is distributed in the hope that it will be useful, but without any warranty; without even the implied warranty of merchantability or fitness for a particular purpose. Please refer to the the GNU General Public License included with this code repository (`gnu_license.txt`) or see `https://www.gnu.org/licenses`.

3 Matlab files

Matlab is used to implement the simplified model (SM) and the general model (GM). The files are structured as main scripts, (sub)scripts executed by main scripts, functions, and MAT-files containing variable definitions. The file `exportSynthetic.m` is used to compare the SM and GM in a variety of test cases, while `runPT.m`, `runTD.m` and `runPTD.m` determine the correct parameters in GM based on laboratory data.

All files have been tested with a standard Matlab 2017a installation running on Windows 10.

3.1 Files that use measured data

In order to optimize the values of the various parameters in the SM as applied to the PCE→TCE, TCE→*c*DCE and PCE→TCE→*c*DCE degradation sequences, three scripts are used: `runPT.m`, `runTD.m` and `runPTD.m`. These scripts require the following files:

- `readReadData.m`
- `createParamStruct.m`
- `generateSimpleKappas.m` (a script)
- `tidyLatexMatrix.m`
- `to_optimize_reduced.m`

- to_optimize.m
- ode_isotopomere.m
- plotIsotopes.m
- input_data\Lab_Data.xlsx (measured data used as input)

Upon execution of runPT.m, runTD.m and runPTD.m, exportData.m can be executed to save and export the modeling results. exportData.m requires:

- exportExperiment.m
- PT.mat
- TD.mat
- PTD.mat
- brandnewkappas.mat
- export_fig\ (external Matlab package)
- *Ghostscript* (external package, see Section 3.3.18)
- nashsutcliffe.m

3.2 Files that model synthetic cases

exportSynthetic.m compares the performance of the SM and GM. It uses:

- input_data/SimulDiffEpsilons_2Simulate.xlsx
- compareMgMs.m
- calcCompMetric.m
- calcEpsilonRayleigh.m

3.3 Description of individual files

3.3.1 runPT.m

A Matlab script that performs a fit of selected parameters in the SM to experimental data. It reads in δ^{13}C and δ^{37}Cl data from lab experiments involving the reductive dechlorination of chlorinated ethenes by the bacteria *Sulfurospirillum spp.* A parameter structure is created and optimized to the data using selected parameters with the standard Matlab function fminsearch.m. This script uses PTE and TCE data.

3.3.2 runTD.m

As for `runPT.m`, but using TCE and *c*DCE data.

3.3.3 runPTD.m

As for `runPT.m`, but using PCE, TCE and *c*DCE data.

3.3.4 readRealData.m

A Matlab function that returns: δ^{13}C and δ^{37}Cl data from the file `input_data\Lab_Data.xlsx` or another specified file, a parameter structure

Usage:
```
[RealData, deltasGoal, Params0] =
readRealData(ExpType, FileName, MakePlot)
```

Inputs:

- `ExpType`: selector of experimental data; 'PT' = PCE→TCE, 'TD' = TCE→*c*DCE, or 'PTD' = PCE→TCE→*c*DCE
- `FileName`: (optional) string argument for defining an alternative experimental data file instead of `input_data\Lab_Data.xlsx`.
- `MakePlot`: (optional) logical argument which, if true, will plot the experimental data.

Outputs:

- `RealData`: table structure containing the experimental data.
- `deltasGoal`: structure array of structure arrays containing the experimental data and weights.
- `Params0`: structure array of initial values of selected model parameters.

Usage example:
`readRealData('PT', [], true)` returns PCE & TCE experimental data from the standard file and plots the data in a new window.

3.3.5 generateSimpleKappas.m

A Matlab script that generates κ matrices (see Equation 2 in the main manuscript) for the dechlorination reactions. The script also creates LaTeX formatted output for these matrices.

3.3.6 createParamStruct.m

A Matlab function that returns or updates a model parameter structure or double array. If no argument is given, a structure array with `NaN` values for all parameters is return. If a structure is given as the argument, the function returns a double array of the values of the parameters.

Usage:
```
S = createParamStruct(x)
```

Inputs:

- `x`: optional parameter structure input to be updated.

Outputs:

- `S`: parameter structure.

3.3.7 tidyLatexMatrix.m

A simple Matlab function that ensures correct formatting of the LaTeX output from `generateSimpleKa`[illegible]

3.3.8 to_optimize.m

A Matlab function to be optimized using `fminsearch`.

Usage:
```
[Cost, Y, deltas, T, Ctot] = to_optimize(x, t,
 KappasP2T, KappasT2C, Ps, Ws,deltasGoal)
```

Inputs:

- `x`: Existing parameter structure
- `t`: Time since beginning of experiment (seconds)
- `KappasP2T`: Degradation rate constant matrix (PCE→TCE)
- `KappasT2C`: Degradation rate constant matrix (TCE→cDCE)
- `Ps`: Structure of symbolic fractionation factors for C and Cl.
- `Ws`: Weights for fractionation factors.
- `deltasGoal`: target isotopic ratios / concentrations and their weights

Outputs:

- `Cost`: measure of weighted error in model vs. experimental data

- `Y`: calculated concentrations
- `deltas`: computed δ^{13}C and δ^{37}Cl
- `T`: time delay
- `Ctot`: calculated concentrations

3.3.9 to_optimize_reduced.m

A Matlab function that serves as a wrapper for `to_optimized.m`.

Usage:
```
[Cost, Y, deltas, T, Ctot] =
 to_optimize_reduced(xReduced, x0Full, WhichReduced,
 OptimizeLimited, t, KappasP2T, KappasT2C, Ps, Ws, deltasGoal)]
```

Inputs and outputs similar to `to_optimized.m` *with additional inputs:*

- `xReduced`: Reduced parameter structure
- `x0Full`: Initial parameter values
- `WhichReduced`: Logical array indicating which parameters to optimize
- `OptimizeLimited`: Logical array for limiting subset of experimental data in optimization

3.3.10 ode_isotopomere.m

A Matlab function that returns the time-dependent first-order ODE to be optimized. Used as part of an anonymous function by `to_optimize.m`.

Usage:
```
dy = ode_isotopomere(y, KappasP2T,
 KappasT2C, muDec, muMax, Yi, Km, Ws)
```

Inputs:

- `y`: Dependent variables of the ODE system
- `KappasP2T`: Degradation rate constant matrix (PCE$\rightarrow$TCE)
- `KappasT2C`: Degradation rate constant matrix (TCE$\rightarrow$cDCE)
- `muDec`: Biomass decay rate constant

- `muMax`: Maximum bacterial growth rate for Monod kinetics (refer to Equations 9 & 10 in the main manuscript); muMax(1) used for PCE→TCE, muMax(2) for TCE→cDCE
- `Yi`: biomass yield (refer to Equation 10 in the main manuscript); Yi(1) used for PCE→TCE, Yi(2) for TCE→cDCE
- `Km`: half-saturation constant (refer to Equations 9 & 10 in the main manuscript); Km(1) used for PCE→TCE, Km(2) for TCE→cDCE

Outputs:

- `dy`: temporal derivatives of all dependent variables

3.3.11 plotIsotopes.m

A Matlab function that creates a 4-pane plot of: concentrations vs. time, δ^{13}C vs. time, δ^{37}Cl vs. time, and δ^{13}C vs. δ^{37}Cl (dual-delta plot).

Usage:
`plotIsotopes(Ctot, deltas, T, RealData, Comsol)`

Inputs:

- `Ctot`: Computed concentrations
- `deltas`: Computed δ^{13}C & δ^{37}Cl values
- `T`: Computed times
- `RealData`: Laboratory data
- `Comsol`: (optional) additional data to plot

3.3.12 input_data\Lab_Data.xlsx

An Excel spreadsheet containing time-series of laboratory-measured δ^{13}C & δ^{37}Cl values for PCE, TCE, and cDCE.

3.3.13 exportData.m

A Matlab script that exports simulation results by calling `exportExperiments.m`.

3.3.14 exportExperiment.m

A Matlab function that exports simulation results to `.mat`, `.xlsx`

Usage:
`MfileName = exportExperiment(ExpName, NoDelay, ChangedParams, LoadCompleteKappa`

Inputs:

- `ExpName`: 'PT', 'TD', or 'PTD'
- `NoDelay`: (optional, currently unused) tag for experiments no delay
- `ChangedParams`: (optional, currently unused) list of changed parameters
- `LoadCompleteKappas`: (optional) tag to select κ terms to load (1 = loads complete κ terms, 2 = calls `generateSimpleKappas`)
- `IgnoreRealData`: (optional, currently unused)

Output:

- `MfileName`: path of exported `.mat` file

3.3.15 PT.mat, TD.mat & PTD.mat

Matlab "MAT-files" exported by `exportExperiment.m` containing definition of multiple variables.

3.3.16 brandnewkappas.mat

A Matlab .mat file containing several variable definitions.

3.3.17 export_fig\ (folder)

`export_fig` is a Matlab package that facilitates exporting of figures to various formats. It aims to faithfully reproduce the figure as it appears on screen. The version included here was obtained from `https://github.com/altmany/export_fig` on 19-Dec-2017. The package is written and maintained by: Oliver Woodford (2008-2014) and Yair Altman (2015-).

3.3.18 Ghostscript

Ghostscript is available under a GNU Affero General Public License from `www.ghostscript.com/download/gsdnld.html`. It is required for the use of the `export_fig` package.

3.3.19 exportSynthetic.m

Matlab script used to execute models with selected ϵ (or AKIE) values (see Table S1). No laboratory data is used.

3.3.20 input_data/SimulDiffEpsilons_2Simulate.xlsx

A spreadsheet containing the selected values of ϵ to be modeled with `exportSynthetic.m`.

3.3.21 compareMgMs.m

Matlab script that compares the SM and GM model outputs.

Usage:
```
[CompMetric, EtaMsMax, DualIsoSlope, EpsMs, EpsMg] =
compareMgMs(MgFile, MsFile)
```

Inputs:

- `MgFile`: path of `.mat` file for the general model output
- `MsFile`: path of `.mat` file for the simplified model output

Outputs:

- `CompMetric`: Matlab structure containing various metrics for the comparison of the two models
- `EtaMsMax`: Matlab structure containing values of η (see Table S3) other parameters
- `DualIsoSlope`: Matlab structure containing values of dual isotope slopes and other parameters
- `EpsMs`: Matlab structure containing the input ϵ values in the SM model
- `EpsMg`: Matlab structure containing the input ϵ values in the GM model

3.3.22 calcCompMetric.m

Matlab function that calculates the value of a selected goodness-of-fit or other data comparison metric.

Usage:
```
[metric] = calcCompMetric(observed, modelled, type)
```

Inputs:

- `observed`: the "observed" (reference) data
- `modelled`: the modelled data
- `type`: string to select the comparison method ('NSC', 'RMSE', 'PAAE', 'MAXABSERR', or 'MAXAENORM')

Output:

- `metric`: value of evaluated metric

3.3.23 calcEpsilonRayleigh.m

Matlab function to determine enrichment factors (see Equation S12).

Usage:
```
[EpsCl, EpsC, R2Plot] =
calcEpsilonRayleigh(delta13C, delta37Cl, Ctot)
```

Inputs:

- `delta13C`: δ^{13}C values
- `delta37Cl`: δ^{37}Cl values
- `Ctot`: total concentration of the species

Outputs:

- `EpsCl`: Estimated ϵ value for Cl
- `EpsC`: Estimated ϵ value for C
- `R2Plot`: Data export for plotting

3.3.24 nashsutcliffe.m

Matlab function that calculates the Nash Sutcliffe Efficiency measure (NSE, see Equation 22).

Usage:
```
[ nse ] = nashsutcliffe(observed, modelled)
```

- `observed`: the "observed" (reference) data
- `modelled`: modelled data

Outputs:

- `nse`: Calculated NSE value

4 COMSOL Multiphysics files

4.1 Overview

The simplified model (SM) was also implemented in COMSOL Multiphysics. Each configuration is implemented in a separate file. These files can optionally be linked to Excel using the module "COMSOL LiveLink for Excel".

4.2 Description of individual files

4.2.1 PT.mph

COMSOL model for the SM of the PCE→TCE experiment.

4.2.2 TD.mph

COMSOL model for the SM of TCE→*c*DCE experiment.

4.2.3 PTD.mph

COMSOL model for the SM of PCE→TCE→*c*DCE experiment.

4.2.4 COMSOL_LiveLink_Template.xlsx

Excel template file that can be linked to COMSOL via the LiveLink for Excel modul to facilitate modification of modeling parameters.

5 Credits

All files, except where specified otherwise, were written by Fabian Braun and Alice Badin, and modified and documented by Landon Halloran.
